# Genome wide gene-expression analysis of facultative reproductive diapause in the two-spotted spider mite *Tetranychus urticae*

**DOI:** 10.1186/1471-2164-14-815

**Published:** 2013-11-21

**Authors:** Astrid Bryon, Nicky Wybouw, Wannes Dermauw, Luc Tirry, Thomas Van Leeuwen

**Affiliations:** Department of Crop Protection, Faculty of Bioscience Engineering, Ghent University, B-9000 Ghent, Belgium; Institute for Biodiversity and Ecosystem Dynamics, University of Amsterdam, Science Park 904, 1098 XH Amsterdam, the Netherlands

**Keywords:** Thermal hysteresis, Inositol, GPCR, Ice binding, Acari

## Abstract

**Background:**

Diapause or developmental arrest, is one of the major adaptations that allows mites and insects to survive unfavorable conditions. Diapause evokes a number of physiological, morphological and molecular modifications. In general, diapause is characterized by a suppression of the metabolism, change in behavior, increased stress tolerance and often by the synthesis of cryoprotectants. At the molecular level, diapause is less studied but characterized by a complex and regulated change in gene-expression. The spider mite *Tetranychus urticae* is a serious polyphagous pest that exhibits a reproductive facultative diapause, which allows it to survive winter conditions. Diapausing mites turn deeply orange in color, stop feeding and do not lay eggs.

**Results:**

We investigated essential physiological processes in diapausing mites by studying genome-wide expression changes, using a custom built microarray. Analysis of this dataset showed that a remarkable number, 11% of the total number of predicted *T. urticae* genes, were differentially expressed. Gene Ontology analysis revealed that many metabolic pathways were affected in diapausing females. Genes related to digestion and detoxification, cryoprotection, carotenoid synthesis and the organization of the cytoskeleton were profoundly influenced by the state of diapause. Furthermore, we identified and analyzed an unique class of putative antifreeze proteins that were highly upregulated in diapausing females. We also further confirmed the involvement of horizontally transferred carotenoid synthesis genes in diapause and different color morphs of *T. urticae*.

**Conclusions:**

This study offers the first in-depth analysis of genome-wide gene-expression patterns related to diapause in a member of the Chelicerata, and further adds to our understanding of the overall strategies of diapause in arthropods.

**Electronic supplementary material:**

The online version of this article (doi:10.1186/1471-2164-14-815) contains supplementary material, which is available to authorized users.

## Background

The seasonal pattern in temperate regions poses a major challenge for arthropod development and survival, and as a result the reproduction and growth of poikilothermic organisms are largely limited to the warmer part of the year. In addition, specific mechanisms have evolved to survive the extreme climatic conditions of winter. One of those mechanisms is a developmental arrest, often considered as the most distinctive characteristic of diapause in insects and mites. In many species, diapause is limited to one particular life stage, and examples have been documented varying from the embryonic stage to larval, pupal and adult stages [1]. Besides developmental arrest, diapause is characterized by a suppression of metabolism [2], altered behavior [3], increased stress tolerance [4] and increased energy reserves [5]. To overcome injury caused by low temperatures, diapausing species can also synthesize cryoprotectants like polyols [6, 7], heat shock proteins [4, 8], and thermal hysteresis proteins [8, 9]. At the molecular level, it is clear that diapause is not simply characterized by a decreased expression of a large number of genes, but that the climate-induced expression of a specific set of genes is crucial to regulate and accomplish the physiological adaptations mentioned above [1].

In spider mites (Acari: Tetranychidae), diapause is limited to the egg or female adult stage, depending on the species [3]. Two varieties of true diapause, i.e. aestival and hibernal diapause have been described, of which the latter is by far the best studied. In the two-spotted spider mite *Tetranychus urticae*, a hibernal facultative reproductive diapause allows adult female mites to survive winter [10]. Since this spider mite species is a highly polyphagous and difficult to control plant pest [11, 12], winter survival has important consequences for crop protection in temperate climates. Therefore, within the group of Acari, diapause is best studied in this species, mainly on the level of behavior and to some extent on the level of biochemistry [3, 13]. Adult female spider mites initiate diapause in response to the reduction of day length during juvenile development. The diapausing females of the green color morphs are characterized by a deeply orange color and suppression of the ovarian development [14]. Next to photoperiod, other factors influence the onset of diapause such as temperature, food quality, food quantity [10] and predation risk [15, 16]. The incidence of diapause can vary greatly between the predominant natural color morphs, red and green, and different *T. urticae* populations. In some populations of the red color morphs, a high percentage of the population enters into diapause after stimulus, while others lack the ability [17]. For the green form of *T. urticae*, this variability is profoundly examined and appears to be dependent on light:day regime [18, 19], the presence of host plants during hibernation period [20] and genetic variability [18, 21]. A recent classical genetic study reveals that in some populations, a single recessive allele is responsible for the non-diapause phenotype [22]. Next to the change in color and developmental arrest, diapause in *T. urticae* is characterized by various biological and physiological changes. A morphological difference between diapause and non-diapause forms is found in the structure of the integumentary striae on the dorsal surface of the cuticle [23], that significantly reduce the evaporative surface and thus the rate of evaporation [24]. At the physiological level, it is known that diapausing mites deposit fat in the body tissues [25] and further limit transpiration through the cuticle by closing the stigmata [26]. Recently, it was experimentally confirmed that *T. urticae* diapausing females show an increased cold hardiness, as the supercooling point of acclimated diapausing female mites is 6°C lower than non-diapausing females [27].

Diapause in *T. urticae* is also accompanied by a number of behavioral changes: mites almost completely cease feeding, leave the host plant in search of hibernation sites and mate immediately after the last molting stage (teliochrysalis), but do not lay eggs [3]. At the time of color change to the typical orange forms, the hindgut is emptied and mites become positively geotactic [28] and negatively phototactic to find shelter [29, 30].

Despite the fact that a number of studies have addressed the changes in morphology, physiology and behavior of diapausing *T. urticae*, the knowledge about the molecular modifications underlying and associated with diapause in spider mites is very limited. Recently, the draft genome of *T. urticae* was reported and a high quality annotation of genes is available [31]. Exploiting this annotated genome sequence and a previously developed whole genome gene expression micro-array platform [12], we studied gene expression changes during diapause in *T. urticae*. Such genome wide transcriptional changes associated with diapause were previously studied in insect species such as the flesh fly *Sarcophaga crassipalpis*[32] and compared with the nematode *Caenorhabditis elegans* and the fruit fly *Drosophila melanogaster*. This comparison reveals that there may be diverse molecular mechanisms, but also that a number of adaptations related to diapause are broadly conserved. In this study, we extend the knowledge on molecular mechanisms underlying diapause in arthropods to the spider mite *T. urticae*, a member of the Chelicerata that diverged more than 450 Mya from other arthropod lineages such as insects and crustaceans [33].

## Methods

### Mite rearing

**Figure 1:**
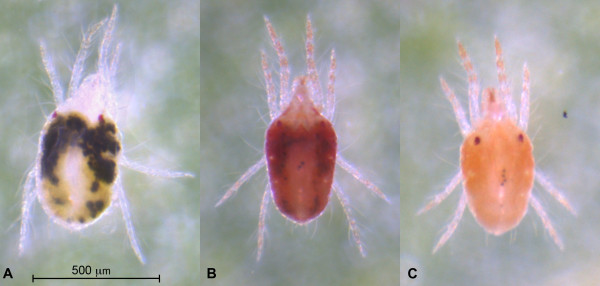
**Different types of body coloration of adult** ***T. urticae*** **females. (A)** Green morphs (LS-VL strain), **(B)** red morphs (Tu-SB9 strain) and **(C)** diapausing forms of green morphs displaying the typical orange color (LS-VL strain).

The green strain LS-VL of *T. urticae* was originally collected in October 2000 near Ghent [34] and was ever since maintained on potted kidney bean plants *Phaseolus vulgaris* L. var. Prelude in controlled conditions at 24 ± 0.5°C and 60% RH with a 16:8h light:dark photoperiod (standard incubation conditions). For induction of diapause, 500 LS-VL adult females were transferred to a single bean plant to allow the deposition of eggs and were kept in standard conditions for 4 days until the larvae hatched. Subsequently, this bean plant and 7 other uninfected plants were held under diapause inducing conditions at 17 ± 0.5°C, 80% RH with 8:16h light:dark photoperiod. After 3 weeks, diapausing females with a distinguishing orange color (Figure 1C) and non-diapausing females (Figure 1A) were collected for RNA extraction. Other strains (London, MR-VL and Tu-SB9) used in this study for qPCR experiments were kept in the same standard incubation conditions. The London strain is a green morph of *T. urticae* that was originally collected in the Vineland region, Ontario, Canada, and originates from the culture used in the *T. urticae* genome project [31]. MR-VL is a red morph and a well characterized multi-resistant strain [35]. Tu-SB9 was collected in Crete, Greece in 2006 and is a red morph (Figure 1B) of *T. urticae*[36].

### RNA preparation for microarray experiments

In order to extract total RNA, four replicates of 250 adult female mites with distinguishing characteristics of diapause (deep orange color, Figure 1C) were collected and homogenized according to the protocol of RNeasy mini kit (Qiagen). The 4-fold replicated control groups consisted of 100 adult females that did not enter diapause under similar diapause inducing conditions (Figure 1A). An additional washing step with one volume of chloroform:iso-amylalcohol (24:1) was performed to decrease the amount of pigments in the supernatant [37]. The quality and quantity of the total RNA was analyzed by a NanoDrop ND-1000 spectrophotometer (NanoDrop Technologies) and by running an aliquot on a 1% agarose gel.

### RNA preparation for qPCR

For qPCR experiments, total RNA was extracted from 3 replicates of 100 adult females of the strains LS-VL, London, MR-VL and Tu-SB9. After homogenization, RNA was extracted according to the protocol of the RNeasy mini kit (Qiagen). The quality and quantity of total RNA was analyzed as abovementioned.

### Microarray construction

A custom Sureprint genome wide G3 Gene Expression 8x60K microarray (GEO Platform accession GPL16890) was designed using the Agilent eArray platform (Agilent Technologies) based on the *T. urticae* gene annotation file frozen in April 2011, including coding sequences of 18,217 predicted unigenes, as previously described [12, 38]. The slide layout consisted of eight arrays per slide.

### Microarray preparation, hybridization and analysis

One hundred nanograms of RNA of non-diapausing (4 replicates) and diapausing mites (4 replicates) were used to generate Cy3- and Cy5-labeled cRNA respectively, using the Agilent Low Input Quick Amplification labeling kit (version 6.5, Agilent Technologies). RNA spike-in controls (Agilent Technologies) were added to diluted aliquots of RNA samples before cRNA synthesis. The labeled cRNA was purified with the RNeasy mini kit (Qiagen). The dye content and concentration of cRNA was measured by NanoDrop ND-1000 spectrophotometer (NanoDrop Technologies). Cy3- and Cy5-labeled cRNAs were pooled and hybridized using the Gene Expression Hybridization Kit (Agilent Technologies) for 17 h in a rotating hybridization oven at 10 rpm and 65°C. After hybridization, slides were washed using the Gene Expression Wash Buffer kit (Agilent Technologies), treated with Stabilization and Drying solution (Agilent Technologies), protected by an Ozone-Barrier cover (Agilent Technologies) until scanned by an Agilent Microarray High Resolution Scanner with default settings for 8 × 60K G3 microarrays. Data were normalized by the Agilent Feature Extraction software version 10.5 (Agilent Technologies) with default parameter settings for gene expression two-color microarrays (protocol GE2_107_SEP09) and transferred to GeneSpring GX 11.0 software (Agilent Technologies) for further statistical evaluation. Next, probes were flag filtered (only probes that had flag-value ‘detected’ in 50% of the replicates were retained) and linked to the most recent annotation file (September 2011, [39]) using the ‘Create New Gene-Level Experiment”-option. Genes with a Benjamini-Hochberg false discovery rate (FDR) corrected p-value < 0.05 and with an absolute fold change (FC) ≥ 2 were considered as differentially expressed. The microarray data reported in this paper have been deposited in the Gene Expression Omnibus (GEO) (accession number: GSE48858).

### Functional analysis

Differentially expressed genes were exported to Blast2GO software v.2.6.3 [40]. This tool enables homology searches based on Protein Basic Local Alignment Search Tool (BLASTp) against the NCBI non-redundant protein database using an E-value cut-off of 1e^-15^. Subsequently, Blast2GO mapping performs different steps to link all best BLAST hits to information stored in the Gene Ontology (GO) database. All these mapping results are associated to an Evidence Code which provides information about the quality of mapping. After mapping, the results were subjected to GO annotation whereas GO terms were selected from the GO pool with a threshold of 1e^-15^ and assigned to the sequences. Further annotation was done using InterPro annotation in Blast2GO and corresponding GO terms were transferred to the sequences and merged with already existent GO terms. For the complete genome, the functional analysis stopped here. But for the genes that were differentially expressed in the microarray, GO terms were modulated using the Augment annotation tool by Annex [41] followed by GO-Slim which is a reduced version of the Gene Ontology database that contains a selected number of relevant nodes.

### Gene ontology terms enrichment analysis

Within Blast2GO, an analysis was performed on gene function information, by the statistical assessment of GO term enrichment in a group of genes of interest when compared with a reference group i.e. to assess the functional differences between two sets of functional annotations. This was integrated in Blast2GO by Gossip [42] that performs a Fisher’s Exact test in combination with a Benjamini-Hochberg False discovery rate (FDR) correction for multiple testing. The option “Two-Tailed” was selected to perform a test which considers both directions as extreme and this translates to over- and under-represented Gene Ontology functions in the test-set compared to a reference set. The result of this test returns a list of significant GO terms ranked by p-values. GO terms with a Fisher Exact test FDR corrected p-values < 0.01 were considered as statistically significant and in order to reduce the size of the result-set to existing, statistically significant, child GO terms, the function “reduce to most specific terms” was used. This function enables to simplify the outcome of GSEA in case of a very large list of enriched GO terms by removing parent terms of already existing, statistically significant, child GO terms that represent the same functional concept but at different levels of specificity.

To identify statistically over-represented functions in our dataset, a Fisher’s exact test was carried out with the selected differentially expressed genes compared to the completely functional annotated genome of *T. urticae.* Between the sets of up-and down-regulated genes, a second enrichment test was performed to detect different biological roles of these sets during diapause.

### Microarray confirmation by qPCR

In order to validate the microarray results, gene specific primers were designed for 13 differentially expressed *T. urticae* genes (7 up- and 6 down-regulated genes) using Primer 3 v0.4.0. [43]. *T. urticae* genes coding for actin, ubiquitin and RP49 were used as housekeeping genes after being tested for their suitability as reference genes by comparing expression patterns between diapausing and non-diapausing forms (Additional file 1). Total RNA was extracted as described above and cDNA was synthesized with 2 μg of total RNA using the Maxima First Strand cDNA synthesis kit for RT-PCR (Fermentas Life Sciences). Three biological and two technical replicates were used to conduct these experiments and no-template-controls were added to exclude sample contamination. All qPCR reactions were carried out with the thermal cycler Mx3005P (Stratagene). Reactions were prepared with Maxima SYBR Green qPCR/Master Mix following the manufacturer’s instructions (Fermentas Life Sciences). The reactions were run with the following protocol: initial denaturation at 95°C for 10s followed by 35 cycles of 95°C for 15s, 55°C for 30s, 72°C for 30s. At the end of these cycles, a melting curve (from 55°C to 95°C, 1°C per 2s) was generated to confirm the absence of non-specific amplification. Standard curves were constructed for every primer pair using different cDNA dilutions to calculate the primer specific amplification efficiency. These efficiencies were incorporated in calculations of the expression values. The housekeeping genes with the highest efficiency and constant Ct values were selected and the obtained Ct values of these genes were used for normalization. Analysis of qPCR results was performed according to Pfaffl [44], producing relative expression values of the target gene. Significant differences in gene expression of the target gene were tested with pairwise fixed reallocation randomization [45].

### *T. urticae* antifreeze proteins (AFPs) analysis

Gene expression analysis revealed the upregulation of 14 genes belonging to the same hypothetical protein family. A tBLASTn analysis (E-value cutoff = 1e^-5^) using these hypothetical proteins as queries was conducted against the *T. urticae* genome (http://bioinformatics.psb.ugent.be/orcae/overview/Tetur). Gene models were refined or created on the basis of homology, RNA-seq and/or EST support and RT-PCR. A BLASTp search, using these hypothetical proteins as queries, was performed against the NCBI nr protein sequence database to detect homologues in other organisms. Identity and similarity matrices were calculated using MatGAT 2.0 [46] while an alignment was created using MUSCLE [47]. *T. urticae* AFP structures were predicted using the Phyre^2^ server [48], in order to create a 3D model for the query sequence, and further edited in Swiss-Pdb viewer [49]. The top match produced by Phyre^2^ is the protein sequence that showed the highest raw alignment score with the query sequence and is based on the number of aligned residues and the quality of alignment. This first match was selected to fit our protein to a 3D model. SignalP 4.0 was used for prediction of signal peptides of AFP protein sequences using default cutoff values for eukaryotes [50].

The LS-VL strain was used for the additional qPCR experiments assessing the effect of temperature on expression levels of *T. urticae* AFP genes. For the samples of 24°C, adult female mites were collected from the culture maintained at standard conditions. The group which experienced a cold shock consisted of female mites that were maintained on a leaf disk at 5°C during 7 days. Subsequently, RNA extraction and cDNA preparation were carried out as abovementioned.

## Results and discussion

### Micro-array analysis of diapausing *T. urticae* females

A laboratory strain (LS-VL) of *T. urticae* was grown under environmental conditions that induce diapause (light:dark regime of 8:16h at a temperature of 17°C). Under these conditions, approximately 10–30% of the population entered diapause. This made it possible to select and collect both diapausing and non-diapausing mites with a common genetic background that developed under identical environmental conditions (Figure 1A and C). The gene expression differences between these two distinctive groups should be mainly linked to diapause and were the foundation of this analysis.

**Figure 2:**
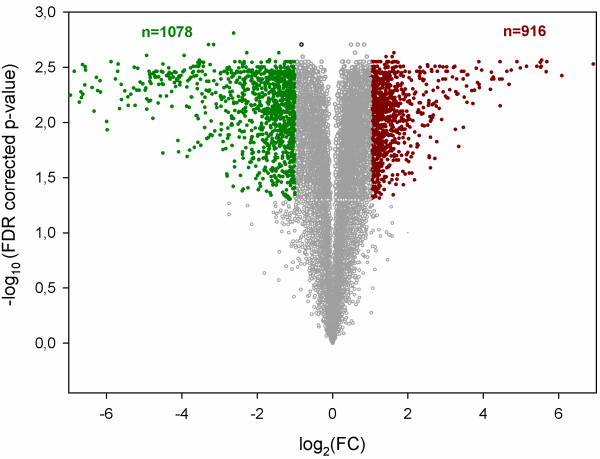
**Volcano plot of differentially expressed genes in diapausing** ***T. urticae*** **females identified by microarray analysis.** The log_10_(FDR corrected p-values) were plotted against the log_2_(FC) in gene expression. Genes upregulated (n = 916) by twofold or more and with a FDR corrected p-value < 0.05 are depicted as red dots, genes that were downregulated (n = 1078) by twofold or more and with a FDR corrected p-value < 0.05 are shown with green dots. All other genes in the array that were not found to be differentially expressed are depicted as grey dots.

We compared gene expression levels between diapausing and non-diapausing mites by analyzing genome-wide microarray experiments, including probes for a total number of 18,217 predicted unigenes [12]. Analysis revealed that 1,994 genes (11% of all predicted genes) were differentially expressed (absolute FC ≥ 2, FDR <0.05) (Figure 2), of which 1078 were downregulated and 916 were upregulated in diapausing females. The expression level of the 20 most upregulated genes varied from a FC of 45 to 407 and included many genes with an unknown function (Additional file 2). Genes with known homologues included a *T. urticae* homologue of 5’ nucleotidase (*tetur16g01680*, FC 67) and a gene encoding a protein with a thioredoxin domain (*tetur46g00020*, FC 46). Among the 20 most downregulated genes in diapausing mites, genes with known functions and homologues included six vitellogenin genes (*tetur39g00700*, *tetur39g00720*, *tetur39g00810*, *tetur43g00010*, *tetur43g00020*, *tetur516g00020*, FC between 172 and 511), one lipase A gene (*tetur09g06700* with a FC of 593), a gene belonging to the peroxidase family (*tetur13g03760*, FC 124), a midA gene (*tetur09g06670*, FC 116), a phosphatidylserine decarboxylase (*tetur12g01500*, FC 102), a glucose dehydrogenase (*tetur03g09330*, FC 99) and a serine protease (*tetur19g00740*, FC 95) (Additional file 2). Noteworthy, the downregulation of vitellogenin genes in diapausing *T. uticae* females was considered by Kawakami *et al.* (2011) [14] as a validation of true diapause.

A confirmation of the microarray experiment was conducted by qPCR. A similar trend was found between fold changes extracted from the complete genome microarray and the mean fold change data obtained by qPCR (Additional file 3).

### Functional analysis and Gene Set Enrichment Analysis (GSEA) of differentially expressed genes in diapausing *T. urticae* females

**Figure 3:**
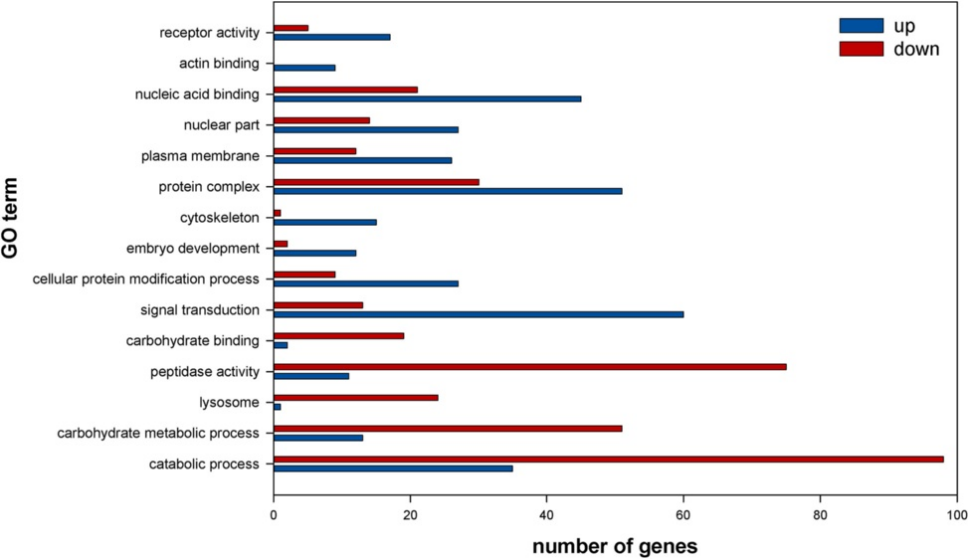
**Gene Ontology (GO) categories with a significant difference between the number of up- and downregulated genes in diapausing** ***T. urticae*** **females.** Red bars show the number of downregulated *T. urticae* genes while blue bars show the number of upregulated *T. urticae* genes for each GO category. The number of upregulated and downregulated genes within each GO category were considered as statistically different when the Fisher Exact test FDR corrected p-value was less than 0.01.

A functional analysis of the differentially expressed genes revealed that 947 genes, 48% of total differentially expressed genes, could be assigned to a GO category. When the complete genome was categorized in a similar way, 6,105 genes, 33% of total number of *T. urticae* genes, were classified in GO terms. Details of the GO classification can be found in Additional file 4. Based on this GO classification, a Gene Set Enrichment Analysis (GSEA) was carried out to screen for an enrichment of the differentially expressed genes compared to the complete genome of *T. urticae* (Fisher Exact test FDR corrected p-values < 0.01, reduced to most specific terms) (Table 1). Notable was the enrichment of gene sets related to oxidoreductase activity (FDR = 3.46e^-05^), negative regulation of multicellular organism growth (FDR = 5.77e^-05^), carboxylic ester hydrolase activity (FDR = 5.19e^-04^) and fatty acid synthase activity (FDR = 2e^-03^). These GO terms are indicative of several expected adaptations on the molecular level further discussed below. Other diapause enriched gene sets were the categories of response to acid (FDR = 1e^-03^) and response to wounding (FDR = 2e^-03^) which are both related to stimuli responses. Underrepresented gene sets of the biological processes of translation (FDR = 6.3e^-04^), cell cycle (FDR = 2e^-03^) and RNA processing (FDR = 4e^-03^), indicate the arrest of cell growth and a decrease in protein synthesis. Subsequently, a GSEA was performed to retrieve information on the over- or underrepresentation of the upregulated genes compared to the downregulated genes (Figure 3). Clear differences were observed for a number of categories. For signal transduction, upregulated genes (n = 60) were more represented than downregulated genes (n = 13) (FDR = 1.86e^-11^). The cytoskeleton and actin binding related GO terms were also more abundant in diapausing mites (FDR = 2.13e^-04^ and FDR = 2e^-03^, respectively). On the other hand, the GO category of peptidase activity was overrepresented in the downregulated genes (FDR = 4.13e^-08^) as well as the GO terms of catabolic processes, carbohydrate binding, carbohydrate metabolic process and the cellular component of lysosome (FDR = 4.97e^-04^, 7e^-03^, 1e^-04^ and 3.2e^-04^ respectively) indicating a shutdown of anabolic and catabolic mechanisms in diapausing females. Combining this analysis with available literature and the draft genome sequence of *T. urticae*, we focused our following analysis on specific processes related to diapause in more detail below.

**Table 1:** **Enriched GO categories in diapausing** ***T. urticae*** **females**

Category	GO term	Term	Enrichment	FDR
Molecular function	GO:0016491	Oxidoreductase activity	Over	3.46E-05
Biological processes	GO:0040015	Negative regulation of multicellular organism growth	Over	5.77E-05
Molecular function	GO:0052689	Carboxylic ester hydrolase activity	Over	5.19E-04
Biological processes	GO:0001957	Intramembranous ossification	Over	0.001
Biological processes	GO:0001101	Response to acid	Over	0.001
Biological processes	GO:0009611	Response to wounding	Over	0.002
Molecular function	GO:0004312	Fatty acid synthase activity	Over	0.002
Cellular component	GO:0043202	Lysosomal lumen	Over	0.004
Biological processes	GO:0008202	Steroid metabolic process	Over	0.004
Biological processes	GO:0030574	Collagen catabolic process	Over	0.009
Cellular component	GO:0005654	Nucleoplasm	Under	4.41E-05
Biological processes	GO:0006412	Translation	Under	6.29E-04
Cellular component	GO:0043232	Intracellular non-membrane-bounded organelle	Under	9.91E-04
Biological processes	GO:0007049	Cell cycle	Under	0.002
Biological processes	GO:0006396	RNA processing	Under	0.004
Molecular function	GO:0005488	Binding	Under	0.005
Cellular component	GO:0030529	Ribonucleoprotein complex	Under	0.005
Biological processes	GO:0016044	Cellular membrane organization	Under	0.005

#### Adaptations in feeding and detoxification

A noticeable behavioral change indicating that *T. urticae* is preparing for a facultative reproductive diapause, is the fact that it stops feeding and empties the stomach, before migrating to hibernation shelters [3]. This phenomenon was clearly reflected in the GO analysis. Many genes coding for enzymes involved in feeding, like digestive and detoxifying enzymes, were differentially expressed during diapause. About 50% of the total number of *T. urticae* cysteine peptidases (44 out of 87), showed a significant downregulation. Cysteine peptidases are involved in hydrolysis of peptide bonds using a catalytic cysteine and at present, 72 families of cysteine peptidases are identified [51]. In diapausing mites, the downregulated genes belong to the papain-like (C1A) family (32 genes), the legumain (C13) family (2 genes) and the calpain (C2) family (10 genes) [51] (Additional file 5). Particularly the C1A family is known to be involved in the digestion of food [52, 53] but this family is also implicated in embryogenesis and metamorphosis [54, 55]. In the insects *Praon volucre*[56] and *D. melanogaster*[57], the overexpression of these cysteine peptidases is associated with the termination of diapause to allow to resume digestion [7].

The genome of *T. urticae* harbors a remarkable number of genes encoding for ABC transporters and well-known detoxifying enzymes such as cytochrome P450 monooxygenases (CYPs), glutathione-S-transferases (GSTs), carboxyl/choline esterases (CCEs). This high number of detoxifying enzymes and ABC transporters in *T. urticae* is thought to be the result of lineage-specific expansions and has recently been shown to be involved in feeding, pesticide resistance and host plant adaptation [12, 31, 38]. The effects of diapause were also reflected in the regulation of these specialized genes. Of all 82 CYPs annotated in the genome, 28 were differentially expressed. Interestingly, the majority of differentially expressed CYP genes are upregulated in diapause (18 CYP genes belonging to the CYP2, CYP3 and CYP4 clan, fold change between 2.0-4.1) (Additional file 6). Next to detoxification, CYPs contribute to several vital processes including biosynthesis and degradation of ecdysteroids and juvenile hormones and the metabolism of plant allelochemicals and pesticides [58]. In the silkmoth, *Antheraea yamamai,* a CYP gene (CYP4G15) was highly expressed during diapause in the pharate first instar larvae and was suggested to be involved in the diapause termination pathway [59]. Another dormancy involved CYP is the *daf-9* gene of the nematode *C. elegans*, belonging to the CYP2 clan and resembles steroidogenic and fatty acid hydroxylases as well as xenobiotic detoxifying genes. This gene serves as a central point of developmental control, produces hormonal signals and influences the decision of the nematode to choose between a developmental arrest as a third instar dauer larva or the completion to a reproductive adult [60]. In addition, 10 CYPs were downregulated in *T. urticae* (FC between 2.0-23.2), of which *tetur05g04000* was the most downregulated *T. urticae* CYP gene. The majority of these downregulated CYPs belong to the 389 subfamily that is one of the spider-mite-specific expansions and the regulation of this group appears to be greatly affected by the host plant range [31], suggesting their involvement in xenobiotic detoxification.

Next to CYPs, the expression of *T. urticae* carboxyl/choline esterases (CCEs) was also influenced by the diapausing status of *T. urticae*: 13 genes were downregulated and 18 genes showed an upregulation (Additional file 7). All differentially expressed CCEs in diapausing spider mites, except *tetur19g00850*, belonged to two new clades under the neurodevelopmental root of CCE phylogeny [31]. *Tetur19g00850* encodes the enzyme acetylcholinesterase, one of the best characterized CCEs, which is responsible for the hydrolysis of acetylcholine in the nervous system. This gene was two-fold upregulated in diapausing mites, and expression also appears to fluctuate in the moth *Celerio euphorbiae* during diapausing pupal development during winter [61]. Also in other organisms, cholinesterases have been reported to be involved in diapause. In the brain of giant silkmoths, they have been associated with a reduction of electrical activity during pupal diapause [62]. However, other studies observed changes in the cholinergic system that are more influenced by the growth and development of the nervous system than by the mechanism of diapause induction and termination [63, 64]. The downregulation of 13 CCE genes probably also reflects the decrease in food uptake during diapause.

Twenty-five percent of the 32 *T. urticae* GSTs [31] were downregulated (FC 2–5) while only one (*tetur26g01490*) was upregulated under diapausing conditions (Additional file 8). Three differentially expressed genes were classified into the Delta-class GSTs and 5 belonged to the Mu-class which was previously believed to be vertebrate-specific [31]. One of the downregulated genes, *tetur01g02320* (FC 2), belonged to the Omega-class. Recently, it was suggested that an *Apis cerana cerana* gene belonging to this class displayed protective effects against oxidative stress [65]. In general, GSTs are important in Phase II of the detoxification process, acting by conjugating glutathione to xenobiotics or their derivatives. The lower expression of Delta- and Mu-class GSTs, can be associated with the lack of feeding and the reduced intake of potential xenobiotics. In addition, glutathione is often associated with the removal of reactive oxygen species (ROS) and results in a change of the ratio of reduced glutathione (GSH) over oxidized glutathione (GSSG). This ratio serves as an indicator of oxidative stress and is also influenced by GSTs, which are capable of catalyzing the oxidation of GSH via conjugation. It has been shown for the European corn borer *Ostrinia nubilalis* that GST activity in mitochondria was lower under diapause than under non-diapause conditions indicating a lower GSH/GSSG ratio creating oxidizing conditions [66, 67]. Similar to insects, the downregulation of GST genes in the two-spotted spider mite could indicate the transition of a reducing to an oxidative gut environment under diapause conditions.

The expression of ABC-transporters was also affected and of the 103 ABC genes annotated in the *T. urticae* genome [68]*,* 20 genes displayed a differential expression (Additional file 9). The differentially expressed genes coding for ABC-transporters of class C were all downregulated (FC 2.1-7.3) except for one gene (FC 2). This class of ATP-binding cassette transporters constitutes efflux pumps, also named multidrug resistance associated proteins (MRP), and many proteins of this class have been implicated in reducing the cellular concentration of toxic compounds [68, 69]. The fact that diapausing spider mites do not feed, implies that also the intake of toxic compounds will decrease and downregulation of the ABCC genes could be a consequence of this. On the other hand, in the nematode *C. elegans*, an MRP-1, was detected to mediate the regulation of dauer larvae formation [70], and the precise role of ABCC genes in diapause needs to be further investigated. Four ABC-transporters class G were also differentially expressed. One gene, *tetur17g02510* was upregulated (FC 3.5) and previously identified as a clear orthologue of *D. melanogaster* CG3327, also known as Early gene 23 (E23) [68]. This E23 is a 20-OH ecdysone-induced ABC transporter that is capable of regulating 20E responses during metamorphosis, probably by removing 20E from cells [71]. In *Drosophila* flies, it is also believed that E23 controls the circadian clock in adult flies through ecdysone-mediated expression of the clock gene *vrille*[72]. Although *T. urticae* uses a different molting hormone than flies do (ponasterone A instead of 20E) [31], the upregulation of *tetur17g02510* during diapause and its function at this life stage could be crucial and merits further investigation. In the ABC-transporters class H, six genes showed a differential expression ranging from a downregulation of 5.6 to an upregulation of 4.5. This transporters class was not associated with feeding, but recent studies suggest an involvement in cold hardening in *D. melanogaster*[73], pesticide resistance in *Plutella xylostella*[74] pesticide and lipid transport to the cuticle in *Tribolium castaneum*[75].

Finally, genes encoding for intradiol ring-cleavage dioxygenases (ID-RCDs) were greatly influenced by the condition of diapause. Out of 17 ID-RCD genes, 11 were differentially expressed and all except one, showed a significant downregulation ranging from 2 to 37 times (Additional file 10). These ID-RCDs are among the most compelling cases of horizontal gene transfer in the genome of *T. urticae*, as they are specific for bacteria and fungi, with no known homologues in Metazoa [12, 31]. They seem to have an important role in host plant adaptation, and are also constitutely overexpressed in several highly multi resistant spider mite strains [12]. In bacteria and fungi, they cleave aromatic rings of catecholic substrates, but their precise function in *T. urticae* remains until now unclear [12]. Among detoxifying families, ID-RCDs showed one of the most prominent responses to diapause strongly suggesting an important role in digestion or detoxification.

#### Cryoprotection mechanisms: a potential new type of antifreeze proteins

##### Polyol metabolism

Evolutionary adaptations among arthropods to enter diapause are not limited to those that downregulate the metabolism and energy production, but also include metabolic changes that allow to survive the direct effect of harsh conditions such as life-threatening temperatures. Already in 1957, it was reported that diapausing eggs of the silkworm are accumulating sorbitol and glycerol that serve as cryoprotectants [76]. Thereupon, more polyols and sugars were discovered in various arthropods including mannitol, glucose, ribitol, arabinitol and threitol as well as trehalose and the amino acid alanine [6, 77, 78]. Synthesis of polyols is not exclusively assigned to diapause but is also often attributed to rapid cold-hardening [6, 79]. A biochemical study of diapause in *T. urticae* previously revealed an enrichment of potential cryoprotectants [13]. It was found that during diapause, the level of glucose, maltose, inositol and ribitol increased significantly compared to non-diapausing mites and lowering the temperature to 5°C caused a shift in accumulation to mannitol and sorbitol. Worth mentioning is that the concentration of alanine, together with glutamate, did not decrease during diapause [13]. Alanine is also found to serve as a cryoprotectant in *Gynaephora groenlandicae*[80, 81] and associated with freeze survival in *Enchytraeus albidus*[82] and a freeze tolerant frog *Rana sylvatica*[83].

**Figure 4:**
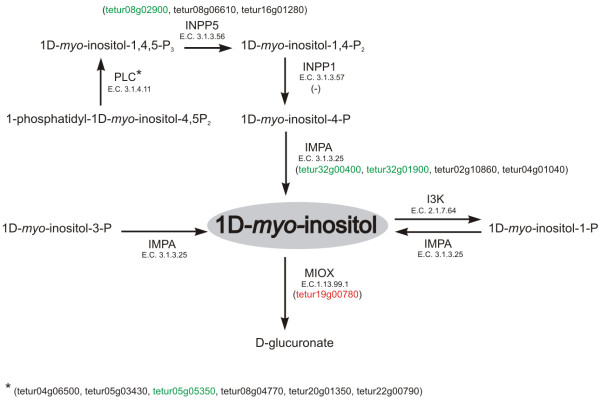
***T. urticae*** **enzymes involved in inositol phosphate metabolism and their expression in diapausing** ***T. urticae*** **females.** The inositol phosphate metabolism pathway was derived from the KEGG metabolic pathway database for *D. melanogaster* (http://www.genome.jp/kegg-bin/show_pathway?ko00562)*.* The name and E.C. number of each enzyme involved in the IPM pathway is listed next to the arrows while accession numbers of *T. urticae* homologues of *D. melanogaster* enzymes involved in IPM are mentioned between brackets (see Additional file 11). *T. urticae* homologues presented with a green font where significantly upregulated (FDR < 0.05, |FC| ≥ 2) in diapausing *T. urticae* females while those shown with a red font were significantly downregulated (FDR < 0.05, |FC| ≥ 2). Abbreviations: INPP1 = Inositol polyphosphate 1-phosphatase, I3K = inositol 3-kinase, IMPA = inositol monophosphatase 3, MIOX = inositol oxygenase, INPP5 = inositol-1,4,5-trisphosphate 5-phosphatase, PLC = phosphatidylinositol phospholipase C.

The results of our gene expression analysis partly support these metabolic changes at the molecular level. Inositol or 1D-*myo*-inositol are intermediates of the inositol phosphate metabolism and some direct key enzymes were differentially expressed (Figure 4, Additional file 11). Inositol monophosphatase (IMPA) dephosphorylates several molecules into 1D-*myo*-inositol and *T. urticae* orthologues of this enzyme, *tetur32g00440* and *tetur32g01900*, were upregulated (FC 2.0 and 2.1 respectively) in diapausing spider mites. Another important enzyme is inositol oxygenase (MIOX) which converts 1D-*myo*-inositol into D-glucuronate and plays a key role in the ascorbate metabolism. Its orthologue, *tetur19g00780*, was 4.3 fold downregulated in diapausing spider mites, possibly to maintain high levels of these cryoprotectants. The enzyme, phosphatidylinositol phospholipase C, PLC, converts 1-phosphatidyl-1D-*myo*-inositol-4,5P_2_ to 1D-*myo*-inositol-1,4,5-P_3_ and its orthologue *tetur05g05350* was upregulated (FC 2.2) during diapause. Subsequently, 1D-*myo*-inositol-1,4,5-P_3_ is dephosphorylated by inositol-1,4,5-trisphosphate 5-phosphatase, INPP5, to 1D-*myo*-inositol-1,4-P_2_. *Tetur08g02900*, one of the genes encoding for this INPP5 enzyme, was upregulated 2.5 times. In conclusion, the differential expression of these enzymes belonging to the inositol phosphate pathway endorses the hypothesis that *T. urticae* synthesizes inositol which is involved in cryoprotection. It was first discovered that inositol accumulates in hibernating adults of beetles [84, 85] and afterwards associated with cold hardening in several insects [85–87]. Also the hibernating adult house spider, which belongs together with *T. urticae* to the Arachnida, was shown to protect against cold-shock damage by the synthesis of inositol [88]. In hydrophilic soil arthropods, the accumulation of *myo*-inositol is essential to increase the osmotic pressure and to maintain their body fluids in a hyperosmotic state. This causes net water uptake from the atmosphere by passive diffusion [89]. Next to inositol related pathways, our analysis also revealed that a maltase gene (*tetur06g01620*) is 4.4 times downregulated, and this could indicate an increase of maltose, which was previously observed by direct measurements in *T. urticae*[13]. Maltose is a disaccharide formed from two units of glucose and its hydrolysis is catalyzed by the enzyme maltase which regenerates glucose levels. This maltose sugar is also enriched in several species during diapause and after cold hardening [90, 91] and is already used as a supplementary cryoprotectant for algae, protozoa [92] and in pretreatment of embryogenic cultures of the maritime pine tree [93].

##### Antifreeze proteins

One of the most striking differentially expressed genes in our analysis consisted of a small family of ‘hypothetical proteins’ (Additional file 2). This family was manually annotated in the genome database of *T. urticae*, and consisted of a set of 20 genes and 2 pseudogenes (*tetur22g03073* and *tetur63g00100*). Of those, only 16 genes were represented by probes on the microarray and 14 of these were upregulated in diapausing spider mites, with fold changes varying between 4 and 164 (Additional file 12). Gene loci are distributed over three genomic scaffolds: 22 (15), 63 (4) and 283 (1) and all genes except *tetur22g03033* are intronless. Genes on scaffold 22 are tightly clustered within a 90 kb region, but the proteins showed only moderate sequence similarity, suggesting their proliferation in the *T. urticae* genome is the result of “ancient” tandem duplication events. *Tetur283g00030*, *tetur63g00030, tetur63g00050, tetur63g00070* and *tetur63g00090* showed very high identity values (96.7% - 100%) with *tetur22g02550*, *tetur22g02640, tetur22g02670, tetur22g02730* and *tetur22g02690*, respectively (Additional file 13). This might suggest a recent duplication event, although the possibility that the genes on scaffold 22 and 63/283 actually represent the same gene due to assembly issues caused by allelic variants cannot be excluded at this stage.

All members of this hypothetical protein family, except *tetur22g02690*, *tetur22g03063* and *tetur63g00090*, have a predicted signal peptide [50] and hence are probably secreted by cells. Strikingly, an InterPro-scan revealed that the majority (16/20) of these hypothetical proteins contained the “Insect Antifreeze Protein” motif (IPR016133 with an E-value between 1e^-04^ and 1e^-06^). A BLASTp search against the non-redundant protein database of NCBI also hit to antifreeze proteins of Coleoptera with a low to moderate E-value (between 1e^-02^ and 1e^-06^). Furthermore, all members from this family were predicted as an insect AFP by AFP-Pred, a recently developed software tool using a “random forest” approach for the prediction of antifreeze proteins [94].

**Figure 5:**
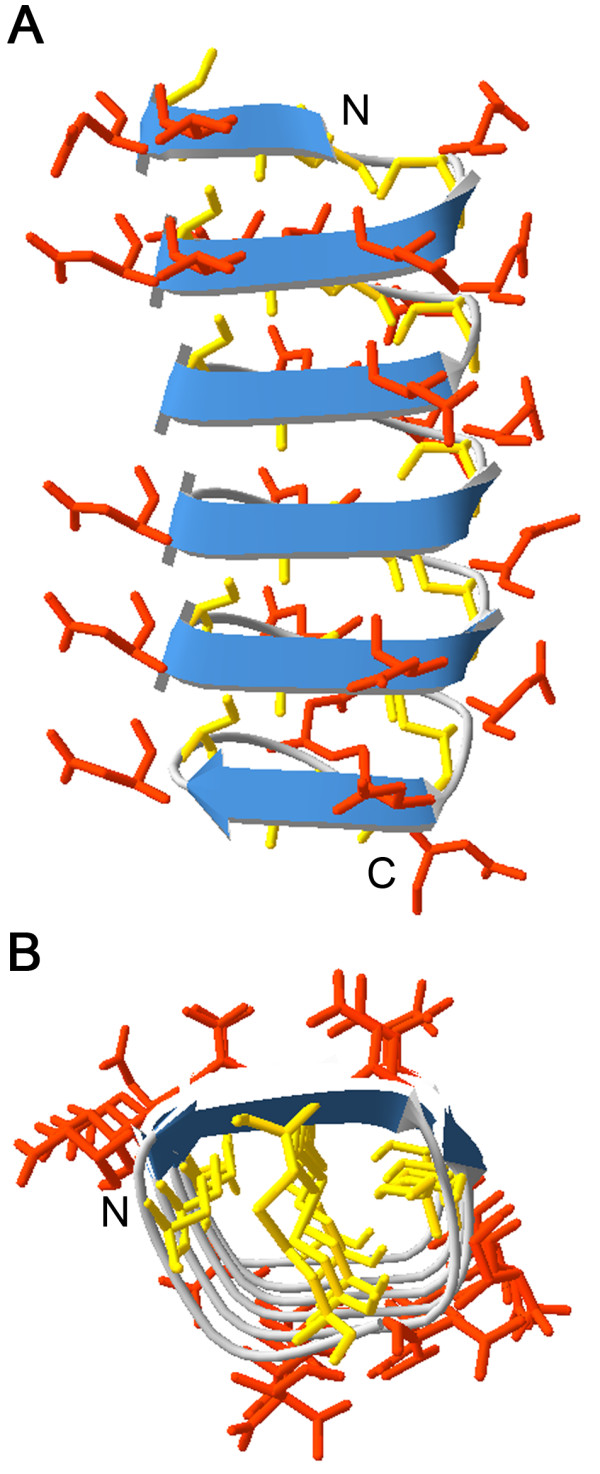
**Ribbon illustration of a putative** ***T. urticae*** **antifreeze protein (AFP).** Side **(A)** and end-on **(B)** view of the predicted 3D-structure of a putative spider mite AFP (tetur22g03033) with β-sheets indicated by blue arrows. Asn, Asp and Thr side-chains are indicated in orange while Cys side-chains are indicated in yellow. The N-and C-terminus of the *T. urticae* AFP structure are indicated with the letters N and C, respectively. The 3D-model for tetur22g03033 was created using the Phyre^2^ server (with pdb-model: d1ezga) [48] and further edited with Swiss PDB viewer [49].

Accordingly, we aligned and compared the *T. urticae* mite sequences with well-studied insect AFP sequences of two beetles: *Dendroides canadiensis* (AAF86362 and AAB94303) and *Tenebrio molitor* (1L1I_A) (Additional file 14). These insect AFPs consist of 78 to 148 amino acids and their cysteine (Cys) content ranges from 15 to 19%, whereas the spider mite sequences measure between 92 and 210 amino acids and consist for 19 to 25% of Cys. In addition, typical insect AFPs are characterized by 7 repeats of 12-or 13-mer repeats (Thr-Cys-Thr-X-Ser-X-X-Cys-X-X-Ala-X) with at least every sixth residue a cysteine [95, 96]. The Cys residues can form stable disulfide bridges throughout the protein and are flanked by Thr residues, which are thought to be responsible for the ice binding sites [97]. In *T. urticae,* the predicted AFPs comprise a continuous repeated sequence (Asn-Cys-Thr-X-Cys-X-X-Cys-X-Asn-Cys-X). It is clear that two of the Cys (at the sixth residue, involved in disulfide bridging) are conserved, but two other Cys are found at positions where in beetles a conserved Ser and Ala residue is found. Furthermore, the conserved Cys are not only flanked by Thr, but a variety of Asp, Asn and Thr. These hydrophilic residues are capable of ice-binding and inhibition of ice crystal growth [98, 99]. The specific arrangement and position of these residues is however fundamental for the activity of AFPs in order to bind to the ice surface with a particular orientation. Hence, all 20 protein sequences were submitted to Phyre^2^ web service for structure prediction. Six out of 20 query sequences (tetur22g02640, tetur22g02690, 22g03033, tetur22g03063, tetur63g00030 and tetur63g00090) had a top scoring match with an insect AFP template structure (pdb: d1ezga) in the Phyre^2^ library. Subsequently, 2D and 3D models for these six *T. urticae* proteins were returned based on this match. For example, the 3D model of tetur22g03033 (53% of the tetur22g02640 protein sequence has been modeled with 97.1% confidence, Additional file 15) showed the typical configuration of cysteine rich insect AFP consisting of six β-strands containing the typical Cys-residues (Figure 5) (at six AA distance) producing disulfide bridges. The important ice-binding amino acids (Asn, Asp and Thr) are located on the outside of the protein. Interestingly, the extra Cys-residues (at three AA distance) specific for mite AFP sequences, are pointing inwards and may form alternative cysteine bridges *in vivo*. The typical ice-binding surface [100–102] was not apparent in the 3D homology model of tetur22g03033, but these outfacing hydrophilic residues might still be involved in ice binding. Recently, divergent tertiary structures were also reported for *Rhagium inquisitor*[103] and snow fleas [104]. In conclusion, sequence alignments, and *in silico* predictions including a homology model strongly suggest that this small family of proteins might represent a new class of arthropod AFPs. Nevertheless, functional expression combined with activity tests using nanoliter osmometry should provide formal evidence of the activity and ice binding properties of these proteins [105–107].

The production of antifreeze proteins is a protective mechanism against the effects of lowered environmental temperatures and freezing [8]. Typically, cysteine-rich insect antifreeze proteins adsorb to seeded ice crystals and inhibit enlargment of these crystals [101] which causes a difference between the freezing temperature and the melting point, a process that is known as thermal hysteresis. Thermal hysteresis antifreeze proteins have also been found in vertebrates, invertebrates, fungi, bacteria and plants [108]. At least fifty species of insects and many terrestrial arthropods [107, 109] are known to produce AFPs and although many have low levels of thermal hysteresis, some insects produce hyperactive antifreeze proteins. The common yellow mealworm beetle, *T. molitor,* contains antifreeze proteins that can account a thermal hysteresis of 5.5°C at a concentration of 1 mg/ml [95]. Comparative results are found in *Hypogastrura harveyi*[110, 111] and *C. fumiferana* who display similar thermal hysteresis properties [108]. The magnitude of thermal hysteresis effect occurring in a species depends not only on the structural type of proteins, but can differ greatly depending on the moment of sampling or the level of acclimatization of insects [111].

**Figure 6:**
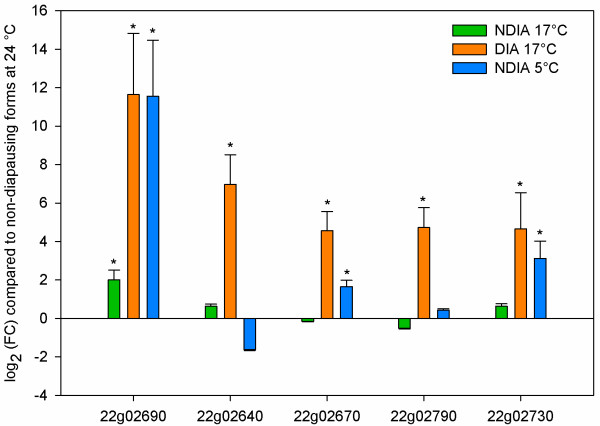
**Expression levels of genes coding for putative** ***T. urticae*** **antifreeze proteins.** qPCR quantification of expression levels of putative AFP genes in *T. urticae*. Green, orange and blue bars represent the relative mean expression in non-diapausing spider mites at 17°C, diapausing spider mites at 17°C and non-diapausing spider mites at 5°C, respectively, relative to expression in non-diapausing mites of the LS-VL strain at 24°C. Error bars represent the standard error of the calculated mean based on three biological replicates. Asterisks indicate significantly differential expressed genes (random reallocation test) compared to the reference condition (green, non-diapausing LS-VL strain at 24°C).

We performed additional qPCR experiments to investigate the association of AFPs with diapause and/or cold stress of 5 typical AFP genes (*tetur22g02690, tetur22g02640, tetur22g02730, tetur22g02790* and *tetur22g02670*). Expression levels were compared between non-diapausing mites at 24°C (standard rearing conditions), and mites that were submitted to a cold shock of 7 days, together with diapausing and non-diapausing mites at 17°C (the conditions of the microarray experiments). Results showed that the expression of these genes was overall more affected by the diapause condition, than the reduction in temperature in *T. urticae*, indicating that for some genes the expression is probably regulated by physiological, rather than environmental changes (Figure 6). All genes were significantly high upregulated in diapausing mites at 17°C when compared to the expression of these genes in active mites at 17°C, confirming microarray results. Only one gene, *tetur22g02690*, was equally high expressed both in diapause mites at 17°C and in mites treated by cold shock. Of the 4 other genes tested, two did respond to cold stress (*tetur22g02670, tetur22g02730*) but with small changes in expression, while *tetur22g02790* and *tetur22g02640* were only upregulated to high levels in diapausing mites.

It was previously shown that the production of antifreeze proteins is not exclusively associated with diapause. In *T. molitor*, *D. canadensis*[112]*, R. inquisitor*[113] and *H. harveyi*[110], antifreeze proteins could be collected from insects that were exposed to cold. In the spruce budworm, transcripts of antifreeze proteins were found in both the first larval instar and in the second diapausing instar [111]. This indicates that both cold hardening and diapause can trigger the production of AFPs in insects, similar to what was shown for *T. urticae* in this study. It was demonstrated previously in *T. urticae* that diapausing forms had a lower supercooling point than non-diapausing forms, even if those active forms were cold acclimated for 10 days at 5 or 0°C [27]. Furthermore, the 5°C cold acclimated active mites showed a lower supercooling point than the not acclimated active mites, indicating cryoprotection caused by cold and diapause, but the involvement of mite AFPs identified in this study was not assessed and should be further studied. Of particular note, homologues of spider mite AFPs were not found in other mite and tick species for which a draft genome or transcriptome is available, such as *Varroa destructor*, *Metaseiulus occidentalis*, *Ixodes scapularis* and *Panonychus citri*.

#### Cytoskeletal organization

The cytoskeleton plays a critical role in the process of cold-acclimatization in arthropods. For *Culex pipiens*, it was reported that two actin genes were highly overexpressed in early diapause [114, 115]. In addition, the upregulation of actin genes was also associated with a redistribution of polymerized actin during exposure to cold in non-diapausing and diapausing mosquitoes. The functional analysis of our enriched GO terms showed that more genes associated with the cellular component of the cytoskeleton and the molecular function of actin binding were upregulated than downregulated during diapause (Figure 3). Of the 15 genes associated with the cytoskeleton, five showed high identity with the myosin heavy chain protein (a BLASTp hit (E-value < 1e^-144^) of *Camponotus floridanus*). Myosin heavy chain (MHC) isoforms are complex, multifunctional contractile proteins with an ATPase activity and are associated with other MHCs and myosin light chains to form the sarcomeric thick filament [116, 117]. Many organisms have several MHC isoforms that fulfill various functions and differ in their expression patterns. They were found to be involved in translocation of actin filaments [118] and are selectively repressed during dissolution of insect skeletal muscles at the end of the metamorphosis of *Manduca sexta*[119]. In crustaceans, it has been shown that the increased expression of MHC was temperature related and could not specifically be ascribed to diapause [120]. More recently, a sequence that matched with a *Drosophila* MHC was found in the diapause-destined *Artemia fransicana* embryo [121]. In *T. urticae,* two genes, *tetur20g00190* and *tetur20g00160*, that are coding for muscle-specific proteins 300 (MSP300) are also upregulated and are associated with the actin microfilament system [122]. A similar muscular protein, MSP20, was also found to be upregulated in the diapausing *P. volucre*[56]. The gene *tetur23g00300* is related to a formin homology 2 domain containing protein (a BLASTp hit (E-value = 8.59e^-133^) with formin homology 2 domain containing 3 of *Homo sapiens*) and is known as an actin-organizing protein. *Tetur23g00300* was upregulated and the GO terms of actin binding and cytoskeleton components were assigned to this gene. In the GO-term pool of actin binding, two genes, *tetur11g04370* and *tetur11g04320*, with a LIM domain were upregulated. These LIM domains are present in many proteins with diverse functions. One of these functions involves a direct or indirect interaction with the actin cytoskeleton. Besides these genes that came up in the GSEA, other cytoskeleton-related genes also showed a differential expression. Four ankyrin related genes, *tetur01g08790, tetur11g02270, tetur15g02730* and *tetur19g00130*, were upregulated, while a cuticular protein, *tetur06g01680*, was downregulated. This is exactly the opposite of what was observed in the proteomic profiling of the aphid parasitoid *P. volucre*[56]. Ankyrin proteins are implicated in coupling integral membrane proteins to the cytoskeleton network which can cause dynamic interactions among cytoskeletal filaments. Moreover, the upregulation of ankyrin-like proteins is linked to the recovery of insects from cold stress [8]. The differential gene expression of genes coding for cytoskeleton-related proteins suggests that a reorganization of these structures might take place during diapause and that the cytoskeleton is considerably distinct from that of non-diapausing spider mites.

#### Carotenoid synthesis

Already in 1974, Veerman [123] detected that diapausing females of *T. urticae* showed a more than two-fold increase in total keto-carotenoid pigment content than the green summer females. Further research demonstrated that carotenoids not only cause the striking color difference between diapausing and non-diapausing females, but also that carotenoids are functionally involved in photoperiodism [124, 125]. The perception of this photoperiod by mites requires the presence of a photoreceptor and was long unknown. Rearing albino strains on carotenoid-containing and carotenoid-free diets demonstrated the potential of vitamin A as photoreceptor [126]. When wild-type females fed on the carotenoid free diets, they could still enter diapause which according to Bosse *et al.* (1996) could be allocated to the transfer of maternal carotenoids to the egg [126]. In this study it was also assumed that the amount of carotenoids present in the egg was not only enough for diapause induction but also for the body coloration of the diapausing female. Recently, Grbić *et al.* (2011) demonstrated that *T. urticae* possesses two horizontally transferred fused carotenoid cyclase/synthase genes and three carotenoid desaturase genes [31]. These genes most likely originate from a Mucorales-related fungal donor and are similar to the pea aphid, *Acyrthosiphon pisum,* carotenoid genes [127]. Varying numbers of diversified carotenoid desaturases have been found in 34 aphid species and the related insect group Adelgidae [128]. Most recently, Cobbs *et al.* (2013) [129] reported the third case of fungal carotenoid biosynthesis gene homologues in gall midges (Diptera: Cecidomyiidae). Several other cases of horizontal gene transfer have been described in arthropods and most of them have their origin in bacteria, particularly in the *Wolbachia* genus. Only few of those transferred genes to arthropods have been proven to be functional [130, 131].

**Figure 7:**
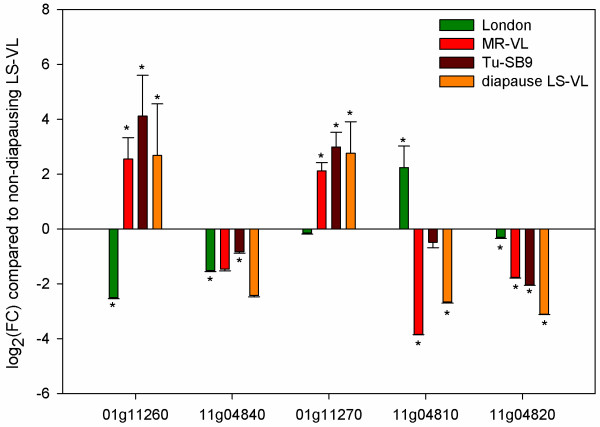
**Expression levels of carotenoid biosynthesis genes in** ***T. urticae.*** qPCR quantification of expression levels of carotenoid biosynthesis genes in *T. urticae*. Green, light red, dark red and orange bars represent the relative mean expression in non-diapausing spider mites of the London strain (green morph), non-diapausing spider mites of the MR-VL strain (red morph), non-diapausing red spider mites of the Tu-SB9 strain (red morph) and diapausing spider mites, respectively, relative to expression in non-diapausing forms of the LS-VL strain (green morph). Error bars represent the standard error of the calculated mean based on three biological replicates. Asterisks indicate significant different expression compared to the reference condition (green, non-diapausing LS-VL strain at 24°C).

In our microarray experiment, four genes involved in carotenoid synthesis were clearly differentially expressed in diapausing females (Additional file 16). *Tetur01g11260* and *tetur11g04840* encoding for the fused lycopene cyclase/ phytoene synthase genes showed an upregulation (FC 6.2) and a downregulation (FC 1.8) respectively. Two out of three genes of the phytoene desaturase genes showed the same trend of regulation. One gene,*tetur01g11270*, displayed an upregulation with fold change of 5.8 and another phytoene desaturase gene, *tetur11g04820*, was downregulated (FC 24.5). Subsequently, we analyzed the expression of this complete set of carotenoid genes by qPCR in two green morphs (LS-VL and London), two red morphs (MR-VL and Tu-SB9) and one diapausing condition of one of the green morphs (LS-VL, 17°C and 8:16 L:D) (Figure 1). All results were compared to one of the green morphs (LS-VL) cultured at 24°C and 16:8 L:D (Figure 7). The expression of the fused lycopene cyclase/ phytoene synthase genes varied depending on the color and physiological state (diapause or not). The cyclase-synthase gene *tetur01g11260* showed a consistent higher expression in diapausing and red morphs, while the cyclase-synthase gene *tetur11g04840* was downregulated in all conditions compared to non-diapausing LS-VL. The expression of the three phytoene desaturase genes resembles the trend of the cyclase/synthase genes, as one gene,*tetur01g11270*, is upregulated in both red morphs and diapausing mites and the other two genes, *tetur11g04810* and *tetur11g04820*, are downregulated in red and diapausing mites. Moreover, the downregulation of *tetur11g04810* was more pronounced: fold changes for the MR-VL red morph and diapausing mites were 14.3 and 6.3 respectively. In the green London morph, this gene was upregulated with a fold change of 4.7 when compared to the green LS-VL morph. From this experiment, it seems that two out of five carotenoid genes are actively involved in the carotenoid synthesis during diapause conditions and in the active red morphs. This data strengthens the general expectation that these genes will enable mites, aphids and gall midges to synthesize their own carotenes, however the functionality of these genes has yet to be determined. Besides this group of horizontally transferred carotenoid genes, genes encoding for GGPP synthase and carotenoid oxygenases were also detected in *T. urticae.* One of these carotenoid oxygenase genes*, tetur06g06440* showed a downregulation in diapausing spider mites which could indicate a shift in beta-carotene molecules.

Previously, the gene regulation of one carotenoid desaturase (*tetur01g11270*) and one fused carotenoid cyclase-synthase (*tetur01g11260*), two of the whole set of five genes, were examined in green morphs, red morphs and diapausing mites by Altincicek *et al.* (2011) [132]. Their expression was subsequently investigated and showed that both genes were upregulated in red morphs in comparison to the green morphs. Diapause conditions had a great influence on the expression, however at the final point of sampling, the green diapause morphs did not display the typical orange color indicative of diapause. Hence, possibly mites in this study had not truly entered diapause, as many populations do not enter it, and results reflect that the conditions to induce diapause are sufficient to trigger the differential expression. Alternatively, pigmentation in the adult morphs might only slowly develop in these populations after the last molt, which has been previously documented [14].

It was suggested that carotenoids and their derivates fulfill many important roles in insect physiology and life history [133]. According to Bosse and Veerman, [126, 134], the photoperiodic induction of diapause requires carotenoids in moths, butterflies, wasps and spider mites. Moreover, the receptor responsible for the photoperiod measurement appears to be an opsin photoreceptor that needs vitamin A, requiring carotenoids for its synthesis [134]. Results suggest that the horizontal transfer of carotenoid synthesis genes may allow spider mites to enter diapause without depending on dietary intake of carotenoids. Another function of carotenoids is to protect arthropods from ultraviolet radiation and oxidative stress [133]. In *T. urticae,* it was confirmed that diapausing females exhibit a lower mortality at different doses of UV than the green summer females [135]. Both these threats are known to seriously damage the cellular components of arthropods. In insects, diapause is often accompanied by oxidative stress [32, 136–138] and besides the protective function of GSTs, an upregulation of carotenoids during diapause in *T. urticae* could increase the chance to survive these harsh conditions.

#### Signal transduction and G-protein coupled receptors

The token stimuli for the two-spotted spider mite to initiate diapause are a decrease in temperature or in the length of the photoperiod. These signals are most likely transmitted through messengers like hormones or other chemical substances in order to initiate the structural and functional changes related to diapause. In this experiment, four genes that were predicted to operate as G-protein coupled receptors (GPCRs) displayed a differential expression. *Tetur14g00960*, *tetur05g00270* and *tetur03g05860* show high similarity with the neuropeptide Y receptor, a tachykinin receptor and neuropeptide F, respectively, and were all upregulated (FC ranging from 2.2 to 7.6). Also one relaxin-like GPCR, *tetur15g00700,* was downregulated (FC 5.1) [139]. Furthermore, the GO enrichment analysis revealed a high number of genes associated with signal transduction (GO:0007165), including a number of kinases/phosphatases and hormone responsive genes (Additional file 17). Interestingly, a number of nuclear receptors were downregulated, including the retinoid X receptor beta, *tetur01g09220*, which has been previously implicated in diapause [140]. In addition, one of the 8 paralogs of *Drosophila* DHR96, *tetur36g00260*, was downregulated in diapausing mites. An increased expression of this paralogue has been previously associated with the response to xenobiotics in *T. urticae*[12]. The downregulation of DHR96 in diapausing mites fits to the overall pattern of a decreased expression of genes associated with a reduced intake of xenobiotics. In *Bombyx mori*, a diapause hormone was the first chemical substance that was identified as a maternal control factor to block progeny development [141]. Subsequently, the GPCR for the insect diapause hormone was also discovered and turned out to serve as an inducer for embryonic diapause [142]. Lately, also other GPCRs belonging to a pyrokinin/pheromone biosynthesis activating neuropeptide family have been found in the European corn borer and were downregulated during larval diapause [143]. The differential expression of the GPCRs found in *T. urticae* might indicate a role in the mechanisms underlying triggering and regulation of diapause.

#### Comparison of diapause responses

The gene regulation and gene expression patterns related to diapause appear to vary considerably among different organisms. Ragland *et al.* (2009) compared arrays of dormant and non-dormant phenotypes of *S. crassipalpis, D. melanogaster* and *C. elegans* and deduced that only a small set of 10 differentially expressed genes were regulated similarly [32]. Even among dipterans, patterns of gene expression were not more similar to each other than to *C. elegans.* This finding could imply that the regulation of diapause is not very conserved. On the other hand, two common physiological responses, i.e. those related to metabolism and stress resistance, were affected similarly during diapause across these three species. In order to investigate to which extent regulation of diapause by *T. urticae* aligns with that of these three species*,* we compared the pattern of the 10 across-species markers genes with that of their homologs in spider mites (Table 2). For 5 of these genes, the expression patterns were similar across the four species. One of those genes, pyruvate carboxylase, is associated with both glycolysis as gluconeogenesis. The other irreversible gluconeogenesis enzyme phosphoenolpyruvate carboxykinase showed a slight downregulation where it displayed an upregulation in the dipterans and *C. elegans*. This variation in gene expression during dormancy i.e. dauer and diapause most likely reflects insects, nematodes and Acari having highly diverged life histories and acquired markedly different life styles during the course of their evolution. Furthermore, the life stage and profundity of diapause varies between these species and is likely reflected in diapause gene regulation.

**Table 2:** **Differentially expressed genes between diapausing and active forms of** ***D. melanogaster, S. crassipalpis,*** ***C. elegans*** **and** ***T. urticae*** ^1^

Gene	FlyBase CG number	***D. melanogaster***	***S. crassipalpis***	***C. elegans***	***T. urticae*** ^2^	***T. urticae***accession number^3^
ftz transcription factor 1	CG4059	down	up	down	up	tetur08g06490 (2e-88)
Histone H2A variant	CG5499	**down** ^4^	**down**	**down**	**down**	tetur11g02430 (2e-65)
phosphoenolpyruvate carboxykinase	CG17725	up	up	up	down	tetur12g00070 (0), tetur27g02510 (0)
mutagen-sensitive 209 PCNA	CG9193	**down**	**down**	**down**	**down**	tetur20g01760 (2e-94)
ubiquitin c-terminal hydrolase	CG3431	**down**	**down**	**down**	**down**	tetur05g03970 (1e-108), tetur189g00020 (9e-109)
ribonucleoside diphosphate reductase small subunit	CG8975	**down**	**down**	**down**	**down**	tetur12g00060 (6e-146), tetur27g02500 (6e-146)
smt3	CG4494	down	down	down	**-**	-
trehalose-6-phosphate synthase 1	CG4104	up	down	down	-	-
pyruvate carboxylase	CG1516	**up**	**up**	**up**	**up**	tetur05g04260 (0)
juvenile hormone epoxide hydrolase 2	CG15102	up	down	up	up	tetur11g03700 (2e-90)

^1^ expression data were derived from [32] and this study.

^2^ genes with a Benjamini-Hochberg false discovery rate (FDR) < 0.05 and with an absolute fold change (FC) > 1 were considered as differentially expressed.

^3^ *T. urticae* accession numbers can be accessed at the ORCAE-database (http://bioinformatics.psb.ugent.be/orcae/), values between brackets represent E-values of best BLASTp hits (E-value < e-60) of *T. urticae* proteins with protein sequences (Flybase CG numbers) of *D. melanogaster* genes.

^4^ bold data reflect gene expression changes in the same direction for all four species.

## Conclusions

In this study, a genome-wide microarray system was used to determine patterns of differential gene expression between diapausing and non-diapausing females of the two-spotted spider mite *T. urticae.* Spider mites are chelicerates, a lineage that diverged more than 450 Mya from other arthropod lineages. In these experiments, RNA was collected from a single population in which approximately 30% of the individuals had entered diapause under the applied inducing conditions. This allowed assessing gene expression differences between samples with a highly similar genetic background and which were reared under identical environmental conditions. GO analysis of differentially expressed genes revealed that many metabolic pathways are affected in diapausing females, especially those related to digestion and detoxification, cryoprotection, carotenoid synthesis and the organization of the cytoskeleton. Some of these transcriptional responses confirmed earlier metabolite studies in spider mites on the molecular level. However, we also discovered previously unknown adaptations, such as the proliferation in *T. urticae* of an unique class of putative antifreeze proteins. These proteins were among the genes most strongly upregulated in diapausing females. Although further validation of their activity is needed, these proteins might be suitable for biotechnological applications related to cryoprotection. Furthermore, we documented differential expression of genes involved in signaling and signal transduction such as G-coupled receptor genes, possibly being the starting point towards understanding the regulation of diapause in this species. Comparison with other genome-wide diapause expression studies suggests that some fundamental changes are conserved, but that overall specific strategies have evolved in different species.

## Electronic supplementary material

Additional file 1: **qPCR primers used in this study.** (DOCX 18 KB)

Additional file 2: **Top 20 of up- and downregulated genes in diapausing** ***T. urticae*** **females.** (XLSX 16 KB)

Additional file 3: **Microarray validation by qPCR.** qPCR validation was performed for 13 genes that were differentially expressed in our microarray experiment (*tetur23g00860* (C1A cysteine peptidase), *tetur03g09330* (glucose dehydrogenase), *tetur26g00570* (low-density lipoprotein receptor), *tetur17g03230* (lipase A), *tetur22g02640, tetur22g02670, tetur22g02690, tetur22g02730*, *tetur22g02790* (antifreeze proteins), *tetur01g11260*, *tetur11g04820* (carotenoid desaturases), *tetur01g11270* and *tetur11g04840* (carotenoid synthases). Error bars represent the standard error of the calculated mean based on three biological replicates. Asterisks indicate significantly differentially expressed genes (random reallocation test) compared to the reference condition (green, non-diapausing LS-VL strain at 17°C). Microarray expression data from this selection of genes are shown next to their qPCR expression data. qPCR primers can be found in Additional file 1. (TIFF 2 MB)

Additional file 4: **Blast2GO data distribution of differentially expressed genes in diapausing** ***T. urticae*** **females and of all protein coding genes in the** ***T. urticae*** **genome.** (DOCX 14 KB)

Additional file 5: **Differentially expressed cysteine peptidases in diapausing** ***T. urticae*** **females.** (DOCX 23 KB)

Additional file 6: **Differentially expressed cytochrome P450 monooxygenases (CYPs) in diapausing** ***T. urticae*** **females.** (DOCX 22 KB)

Additional file 7: **Differentially expressed carboxyl/cholinesterases (CCEs) in diapausing** ***T. urticae*** **females.** (DOCX 21 KB)

Additional file 8: **Differentially expressed glutathione S-transferases (GSTs) in diapausing** ***T. urticae*** **females.** (DOCX 18 KB)

Additional file 9: **Differentially expressed ABC-transporters (ABCs) in diapausing** ***T. urticae*** **females.** (DOCX 18 KB)

Additional file 10: **Differentially expressed intradiol ring-cleavage dioxygenases (ID-RCDs) in diapausing** ***T. urticae*** **females.** (DOCX 18 KB)

Additional file 11: ***T. urticae*** **homologs of** ***D. melanogaster*** **enzymes involved in inositol-metabolism.** (XLSX 11 KB)

Additional file 12: **Differentially expressed antifreeze proteins (AFPs) in diapausing** ***T. urticae*** **females.** (DOCX 18 KB)

Additional file 13: **Percentage identity/similarity between** ***T. urticae*** **AFP protein sequences.** (DOCX 25 KB)

Additional file 14: **Alignment of** ***T. urticae*** **antifreeze proteins (AFPs) with those of** ***T. molitor*** **(pdb: 1L1I_A) and** ***D. canadiensis*** **(AFP1A and AFP7 (GenBank acc. numbers AAB94303.1** **and AAF86362.1, respectively).** AFP protein sequences, without their predicted signal peptides, were aligned using MUSCLE [47] and the resulting alignment was graphically edited using BioEdit version 7.2 [144]. (PDF 78 KB)

Additional file 15: **Predicted 3D structure of a putative** ***T. urticae*** **AFP, tetur22g03033. (best viewed with Swiss-Pdb-Viewer** (http://spdbv.vital-it.ch). (PDB 38 KB)

Additional file 16: **Differentially expressed genes involved in carotenoid synthesis in diapausing** ***T. urticae*** **females.** (DOCX 16 KB)

Additional file 17: **Differentially expressed genes involved in signal transduction in diapausing** ***T. urticae*** **females.** (DOCX 23 KB)

## Abbreviations

ABC: ATP-binding cassette (transporter)
AFP: Antifreeze Protein
CCE: Carboxyl/Cholinesterase
CYP: Cytochrome P450 monooxygenase
FC: Fold Change
FDR: False Discovery Rate
GO: Gene Ontology
GSEA: Gene Set Enrichment Analysis
GSH: Glutathione
GST: Glutathione S-transferase
GPCR: G-protein coupled receptors
ID-RCD: intradiol ring-cleavage dioxygenases
INPP5: inositol-1,4,5-trisphosphate 5-phosphatase
L: D: light:dark
MHC: Myosin Heavy Chain
RH: Relative Humidity
ROS: Reactive Oxygen Species.
