# Supplementary material for: Genome wide gene-expression analysis of facultative reproductive diapause in the two-spotted spider mite Tetranychus urticae
Source: BMC Genomics. 2013 Nov 21;14(1):815. doi: 10.1186/1471-2164-14-815 (PMC4046741; doi:10.1186/1471-2164-14-815)
Supplement: Supplementary file 1 — Additional file 1: qPCR primers used in this study. (DOCX 18 KB) [file 12864_2013_5534_MOESM1_ESM.docx]

Additional File 1

| **Accession number*** | **Function** | **Primer sequence **** | **T_annealing_ (°C)** |
| --- | --- | --- | --- |
| tetur18g03590 | Ribosomal protein 49 | **F:** CTTCAAGCGGCATCAGAGC  **R:** CGCATCTGACCCTTGAACTTC | 62 |
| tetur09g05350 | Actin | **F:** AACCTTCAACACTCCAGCTAT  **R:** GATCTCTACCAGCCAAATCTA | 55.5 |
| tetur03g06910 | Ubiquitin C | **F:** GTCTCCGTGGTGGAATGC  **R:** TTGGATTTTGGCTTTCACG | 60 |
| tetur01g11260 | Carotenoid cyclase/synthase | **F:** TGGTGGATTTGTCCGGTAA  **R:** CCCAACAGAGGTAAATTGATGG | 60 |
| tetur11g04840 | Carotenoid cyclase/synthase | **F:** GCTACGCTCATTTCAAACGG  **R:** GATTAGCCACAAGACGTCCC | 60 |
| tetur01g11270 | Carotenoid desaturase | **F:** CATATGATAGTCCTGGCCCG  **R:** TTTATTTGAAGCAATTTCAATCAGC | 60 |
| Tetur11g04810 | Carotenoid desaturase | **F:** CGAGGATCTGGAAAAGCG  **R:** GTTCAATTTCTCGAGCTAAACATTCTA | 60 |
| tetur11g04820 | Carotenoid desaturase | **F:** TTGGTGAATCTATGGATGACCA  **R:** CAATTTCTTGAGCTAAACGTGTCC | 60.5 |
| tetur22g02640 | Antifreeze protein | **F:** ATTGTGCCAGTTCTAAAGGTTG  **R:** ATTTGCACGATTTGCACAG | 58 |
| tetur22g02790 | Antifreeze protein | **F:** CACACCTTCTACTGGCTCTGG  **R:** CAACCCACACAATTTACACAG | 57 |
| tetur22g02730 | Antifreeze protein | **F:** GATTGTACGAACTGTAAAGATTGTAGG  **R:** CTACTGTATCGTCACAGCCTTG | 57.5 |
| tetur22g02670 | Antifreeze protein | **F:** GATTCTACCGATGGTTCATATTTAC  **R:** TCAGACAGTTTGCACAATTGG | 58 |
| tetur22g02690 | Antifreeze protein | **F:** TGTACCCGATGTAACGATTGTG  **R:** TGCTCTTCTTGGACACTTCTTG | 60 |
| tetur23g00860 | C1A cysteine peptidase | **F:** TCTGATTACATATTCATTCGGAGGTA  **R:** TTATAAATTCCTCCTTTGTAAAACTTG | 58 |
| tetur03g09330 | Glucose dehydrogenase | **F:** TTTGTGTGCTTTTTCACTTTCC  **R:** TGCTAATCGACCTTTTCCATATTC | 60 |
| tetur26g00570 | Low-density lipoprotein receptor | **F:** TTTCAACGATGCTGTAATTTGG  **R:** CTCATCTAATCCTGGGCTAACG | 60 |
| tetur17g03230 | Lipase A | **F:** TTAGACGGACTCTCAGACGTCC  **R:** TCCATTGACAAAACGTCCAG | 60 |

* *T . urticae* accession numbers and their corresponding gene sequences can be found at the ORCAE database (<http://bioinformatics.psb.ugent.be/orcae/overview/Tetur>)

** F= forward primer, R= reverse primer
