# Supplementary material for: Genome wide gene-expression analysis of facultative reproductive diapause in the two-spotted spider mite Tetranychus urticae
Source: BMC Genomics. 2013 Nov 21;14(1):815. doi: 10.1186/1471-2164-14-815 (PMC4046741; doi:10.1186/1471-2164-14-815)
Supplement: Supplementary file 4 — Additional file 4: Blast2GO data distribution of differentially expressed genes in diapausing T. urticae females and of all protein coding genes in the T. urticae genome. (DOCX 14 KB) [file 12864_2013_5534_MOESM4_ESM.docx]

Additional File 4

|  | Microarray data | Genome |
| --- | --- | --- |
| Without blast result | 0 | 17 |
| Without blast hits | 976 | 8935 |
| Only blast results | 79 | 717 |
| Only mapping results | 93 | 2512 |
| Annotated sequences | 947 | 6105 |
