# Supplementary material for: Genome wide gene-expression analysis of facultative reproductive diapause in the two-spotted spider mite Tetranychus urticae
Source: BMC Genomics. 2013 Nov 21;14(1):815. doi: 10.1186/1471-2164-14-815 (PMC4046741; doi:10.1186/1471-2164-14-815)
Supplement: Supplementary file 5 — Additional file 5: Differentially expressed cysteine peptidases in diapausing T. urticae females. (DOCX 23 KB) [file 12864_2013_5534_MOESM5_ESM.docx]

Additional File 5

| **Gene family** | ***T. urticae***  **accession number*** | **Regulation** | **Absolute**  **Fold change** | **Corrected**  **p-value** | **Gene name** |
| --- | --- | --- | --- | --- | --- |
| cysteine peptidase: C1A | tetur02g11420 | down | 83.25 | 0.005 | TuPap-9:Cathepsin B (cathepsin B) |
| cysteine peptidase: C1A | tetur23g00860 | down | 50.37 | 0.007 | TuPap-53:Cathepsin L (cathepsin K precursor) |
| cysteine peptidase: C1A | tetur23g00890 | down | 30.47 | 0.006 | TuPap-57:Cathepsin L-like (cathepsin L) |
| cysteine peptidase: C1A | tetur23g00880 | down | 24.74 | 0.007 | TuPap-30:Cathepsin L (GL17477) |
| cysteine peptidase: C1A | tetur12g01830 | down | 16.59 | 0.005 | TuPap-40:Cathepsin L (cathepsin L) |
| cysteine peptidase: C1A | tetur23g00050 | down | 12.13 | 0.009 | TuPap-29:Cathepsin L (chymopapain isoform II) |
| cysteine peptidase: C1A | tetur02g08840 | down | 9.61 | 0.005 | TuPap-52:Cathepsin L (cathepsin L-like protein; cysteine proteinase) |
| cysteine peptidase: C1A | tetur12g04631 | down | 7.15 | 0.006 | TuPap-39:Cathepsin L (cathepsin L) |
| cysteine peptidase: C1A | tetur01g16463 | down | 6.96 | 0.003 | TuPap-18:Cathepsin-B (cathepsin B) |
| cysteine peptidase: C1A | tetur01g05230 | down | 6.81 | 0.008 | TuPap-20:Cathepsin B (Cathepsin B-like cysteine proteinase precursor) |
| cysteine peptidase: C1A | tetur01g16473 | down | 6.23 | 0.038 | TuPap-28:cathepsin-B (cathepsin B8 cysteine protease) |
| cysteine peptidase: C1A | tetur12g01840 | down | 5.81 | 0.007 | TuPap-51:Cathepsin L (cysteine proteinase) |
| cysteine peptidase: C1A | tetur01g05480 | down | 5.72 | 0.003 | TuPap-17:Cathepsin B (cathepsin B) |
| cysteine peptidase: C1A | tetur04g08380 | down | 4.05 | 0.004 | TuPap-1:Cathepsin O (cysteine proteinase) |
| cysteine peptidase: C1A | tetur09g00600 | down | 3.64 | 0.004 | TuPap-4:Cathepsin B (cathepsin B) |
| cysteine peptidase: C1A | tetur12g01820 | down | 3.31 | 0.003 | TuPap-38:Cathepsin L (cathepsin L2) |
| cysteine peptidase: C1A | tetur123g00050 | down | 3.15 | 0.009 | TuPap-56:Cathepsin L (PREDICTED: cathepsin L1-like) |
| cysteine peptidase: C1A | tetur12g01850 | down | 3.00 | 0.004 | TuPap-35:Cathepsin L (hypothetical protein DICPUDRAFT_28222) |
| cysteine peptidase: C1A | tetur09g04470 | down | 2.94 | 0.008 | TuPap-55:Cathepsin L-like (cathepsin H) |
| **Gene family** | ***T. urticae***  **accession number*** | **Regulation** | **Fold change Absolute** | **Corrected**  **p-value** | **Gene name** |
| cysteine peptidase: C1A | tetur28g01420 | down | 2.93 | 0.005 | TuPap-22:Cathepsin B-like (cathepsin B) |
| cysteine peptidase: C1A | tetur09g04400 | down | 2.92 | 0.008 | TuPap-49:Cathepsin L (cathepsin L) |
| cysteine peptidase: C1A | tetur09g00350 | down | 2.82 | 0.005 | TuPap-32:Cathepsin L (Cathepsin L) |
| cysteine peptidase: C1A | tetur23g01290 | down | 2.79 | 0.009 | TuPap-37:Cathepsin L (cathepsin L) |
| cysteine peptidase: C1A | tetur09g00470 | down | 2.77 | 0.004 | TuPap-33:Cathepsin L (midgut cysteine proteinase, putative) |
| cysteine peptidase: C1A | tetur10g01680 | down | 2.74 | 0.008 | TuPap-54:fibroinase precursor, cathepsin L |
| cysteine peptidase: C1A | tetur13g02490 | down | 2.71 | 0.005 | TuPap-34:Cathepsin L |
| cysteine peptidase: C1A | tetur12g01860 | down | 2.66 | 0.005 | TuPap-41:Cathepsin L (cathepsin l) |
| cysteine peptidase: C1A | tetur24g00280 | down | 2.60 | 0.004 | TuPap-2:Cathepsin B (cathepsin B) |
| cysteine peptidase: C1A | tetur06g03040 | down | 2.49 | 0.013 | TuPap-46:Cathepsin L (cathepsin L) |
| cysteine peptidase: C1A | tetur09g04420 | down | 2.36 | 0.011 | TuPap-50:Cathepsin L (cathepsin L) |
| cysteine peptidase: C1A | tetur06g02930 | down | 2.34 | 0.014 | TuPap-45:fibroinase precursor, cathepsin L (cathepsin L) |
| cysteine peptidase: C1A | tetur24g00270 | down | 2.08 | 0.010 | TuPap-21:Cathepsin B (cathepsin B) |
| cysteine peptidase: C1A | tetur02g14540 | up | 3.04 | 0.009 | TuPap-43:Cathepsin L (cathepsin l) |
| cysteine peptidase: C1A | tetur16g03770 | up | 2.87 | 0.009 | TuPap-44:Cathepsin L (cathepsin l) |
| cysteine peptidase: C1A | tetur16g03680 | up | 2.56 | 0.011 | TuPap-31:Cathepsin L (cathepsin L) |
| cysteine peptidase: C2 | tetur05g04550 | down | 4.25 | 0.015 | TuLeg-4:Legumain (hypothetical protein PANDA_013109) |
| cysteine peptidase: C2 | tetur16g03650 | down | 4.25 | 0.005 | TuLeg-15:Legumain (legumain) |
| cysteine peptidase: C2 | tetur452g00010 | down | 3.65 | 0.012 | TuLeg-19:Legumain (legumain) |
| **Gene family** | ***T. urticae***  **accession number*** | **Regulation** | **Fold change Absolute** | **Corrected**  **p-value** | **Gene name** |
| cysteine peptidase: C2 | tetur06g03540 | down | 3.39 | 0.009 | TuLeg-7:Legumain (legumain) |
| cysteine peptidase: C2 | tetur05g04700 | down | 3.37 | 0.010 | TuLeg-10:Legumain (legumain) |
| cysteine peptidase: C2 | tetur05g04710 | down | 3.36 | 0.009 | TuLeg-8:Legumain (legumain) |
| cysteine peptidase: C2 | tetur60g00020 | down | 3.33 | 0.009 | TuLeg-9:Legumain |
| cysteine peptidase: C2 | tetur16g03670 | down | 2.54 | 0.011 | TuLeg-13:Legumain (legumain) |
| cysteine peptidase: C2 | tetur10g00310 | down | 2.31 | 0.006 | TuLeg-1:Legumain (legumain) |
| cysteine peptidase: C2 | tetur08g06030 | down | 2.22 | 0.015 | TuLeg-16:Legumain (legumain) |
| cysteine peptidase: C13 | tetur03g02400 | down | 11.37 | 0.003 | TuLeg-11:Legumain (legumain) |
| cysteine peptidase: C13 | tetur02g14360 | down | 4.04 | 0.007 | TuLeg-12:Legumain (legumain) |

* *T . urticae* accession numbers and their corresponding gene sequences can be found at the ORCAE database (<http://bioinformatics.psb.ugent.be/orcae/overview/Tetur>)
