# Supplementary material for: Genome wide gene-expression analysis of facultative reproductive diapause in the two-spotted spider mite Tetranychus urticae
Source: BMC Genomics. 2013 Nov 21;14(1):815. doi: 10.1186/1471-2164-14-815 (PMC4046741; doi:10.1186/1471-2164-14-815)
Supplement: Supplementary file 6 — Additional file 6: Differentially expressed cytochrome P450 monooxygenases (CYPs) in diapausing T. urticae females. (DOCX 22 KB) [file 12864_2013_5534_MOESM6_ESM.docx]

Additional File 6

| **CYP Clan** | **CYP family** | ***T. urticae***  **accession number*** | **Regulation** | **Absolute**  **Fold change** | **Corrected**  **p-value** | **Gene name** |
| --- | --- | --- | --- | --- | --- | --- |
| Clan2 | CYP392 | tetur27g00350 | up | 4.15 | 0.0110 | CYP392E8:Cytochrome P450 - CYP392E8 (cytochrome P450, family 2, subfamily j, polypeptide 11) |
| Clan2 | CYP392 | tetur02g14020 | up | 3.24 | 0.0028 | CYP392A8:Cytochrome P450 - CYP392A8 (PREDICTED: steroid 17-alpha-hydroxylase/17,20 lyase-like) |
| Clan2 | CYP392 | tetur02g14330 | up | 3.14 | 0.0028 | CYP392A9v2:Cytochrome P450 - CYP392A9v2 (PREDICTED: steroid 17-alpha-hydroxylase/17,20 lyase-like) |
| Clan2 | CYP392 | tetur47g00090 | up | 3.13 | 0.0028 | CYP392A9:Cytochrome P450 - CYP392A9 (PREDICTED: steroid 17-alpha-hydroxylase/17,20 lyase-like) |
| Clan2 | CYP392 | tetur02g14400 | up | 2.93 | 0.0040 | CYP392A10v2:Cytochrome P450 - CYP392A10v2 (PREDICTED: steroid 17-alpha-hydroxylase/17,20 lyase-like) |
| Clan2 | CYP392 | tetur16g03500 | up | 2.81 | 0.0028 | CYP392A7:Cytochrome P450 - CYP392A7 (PREDICTED: steroid 17-alpha-hydroxylase/17,20 lyase-like) |
| Clan2 | CYP392 | tetur11g00530 | up | 2.77 | 0.0042 | CYP392A6:Cytochrome P450 - CYP392A6 (cytochrome P450 1a) |
| Clan2 | CYP392 | tetur16g03790 | up | 2.77 | 0.0031 | CYP392A10:Cytochrome P450 - CYP392A10 (PREDICTED: steroid 17-alpha-hydroxylase/17,20 lyase-like) |
| Clan2 | CYP392 | tetur06g02820 | up | 2.41 | 0.0067 | CYP392E3:Cytochrome P450 - CYP392E3 (PREDICTED: cytochrome P450 17alpha-hydroxylase/17, 20-lyase-like) |
| Clan2 | CYP392 | tetur27g00330 | up | 2.39 | 0.0082 | CYP392E6:Cytochrome P450 - CYP392E6 (PREDICTED: steroid 17-alpha-hydroxylase/17,20 lyase-like) |
| Clan2 | CYP392 | tetur27g00340 | up | 2.20 | 0.0178 | CYP392E7:Cytochrome P450 - CYP392E7 (PREDICTED: steroid 17-alpha-hydroxylase/17,20 lyase-like) |
| Clan2 | CYP392 | tetur27g01030 | up | 2.18 | 0.0143 | CYP392E10:Cytochrome P450 - CYP392E10 (cytochrome P450 CYP379A1) |
| Clan2 | CYP392 | tetur03g04990 | up | 2.15 | 0.0058 | CYP392D2:Cytochrome P450 - CYP392D2 (PREDICTED: cytochrome P450 17alpha-hydroxylase/17, 20-lyase-like) |
| **Gene family** | **CYP family** | ***T. urticae***  **accession number*** | **Regulation** | **Absolute**  **Fold change** | **Corrected**  **p-value** | **Gene name** |
| Clan2 | CYP392 | tetur20g00290 | down | 2.30 | 0.0043 | CYP392B3:Cytochrome P450 - CYP392B3 (cytochrome P450 1A1) |
| Clan2 | CYP307 | tetur10g03900 | down | 2.22 | 0.0051 | CYP307A1:Cytochrome P450 - CYP307A1 (cytochrome P450 1A) |
| Clan2 | CYP392 | tetur20g03200 | down | 2.04 | 0.0056 | CYP392B1:Cytochrome P450 - CYP392B1 (PREDICTED: cytochrome P450 17alpha-hydroxylase/17. 20-lyase-like) |
| Clan3 | CYP385 | tetur11g05000 | up | 3.01 | 0.0147 | CYP385C2:Cytochrome P450 - CYP385C2 (cytochrome P450) |
| Clan3 | CYP384 | tetur38g00650 | up | 2.88 | 0.0053 | CYP384A1:Cytochrome P450 - CYP384A1 (cytochrome P450. 3a18) |
| Clan3 | CYP385 | tetur05g04000 | down | 23.27 | 0.0041 | CYP385B1:Cytochrome P450 - CYP385B1 (cytochrome P450) |
| Clan4 | CYP386 | tetur11g06070 | up | 3.25 | 0.0200 | CYP386A1:Cytochrome P450 - CYP386A1 (CYtochrome P450 family member) |
| Clan4 | CYP389 | tetur34g00510 | up | 2.97 | 0.0113 | CYP389C1:Cytochrome P450 - CYP389C1 (GE11739) |
| Clan4 | CYP389 | tetur25g02060 | up | 2.15 | 0.0473 | CYP389B1:Cytochrome P450 - CYP389B1 (cytochrome P450) |
| Clan4 | CYP407 | tetur20g00830 | down | 4.78 | 0.0038 | CYP407A1:Cytochrome P450 - CYP407A1 (cytochrome P450) |
| Clan4 | CYP389 | tetur05g02970 | down | 4.74 | 0.0065 | CYP389C4:Cytochrome P450 - CYP389C4 (cytochrome P450) |
| Clan4 | CYP389 | tetur05g02960 | down | 3.91 | 0.0073 | CYP389C3:Cytochrome P450 - CYP389C3 (cytochrome P450) |
| Clan4 | CYP389 | tetur05g02950 | down | 3.80 | 0.0068 | CYP389C2:Cytochrome P450 - CYP389C2 (cytochrome P450) |
| **Gene family** | **CYP family** | ***T. urticae***  **accession number*** | **Regulation** | **Absolute**  **Fold change** | **Corrected**  **p-value** | **Gene name** |
| Clan4 | CYP389 | tetur05g06580 | down | 3.41 | 0.0273 | CYP389C10:Cytochrome P450 - CYP389C10 (PREDICTED: cytochrome P450. family 4. subfamily V. polypeptide 2-like) |
| Clan4 | CYP389 | tetur05g06610 | down | 2.88 | 0.0219 | CYP389C8:Cytochrome P450 - CYP389C8 (cytochrome P450) |

* *T . urticae* accession numbers and their corresponding gene sequences can be found at the ORCAE database (<http://bioinformatics.psb.ugent.be/orcae/overview/Tetur>)
