# Supplementary material for: Genome wide gene-expression analysis of facultative reproductive diapause in the two-spotted spider mite Tetranychus urticae
Source: BMC Genomics. 2013 Nov 21;14(1):815. doi: 10.1186/1471-2164-14-815 (PMC4046741; doi:10.1186/1471-2164-14-815)
Supplement: Supplementary file 7 — Additional file 7: Differentially expressed carboxyl/cholinesterases (CCEs) in diapausing T. urticae females. (DOCX 21 KB) [file 12864_2013_5534_MOESM7_ESM.docx]

Additional File 7

| **Gene family** | ***T. urticae***  **accession number*** | **Regulation** | **Absolute**  **Fold change** | **Corrected**  **p-value** | **Gene name** |
| --- | --- | --- | --- | --- | --- |
| CCE | tetur16g02390 | up | 12.02 | 0.004 | TuCCE41: Carboxyl/cholinesterase |
| CCE | tetur16g02420 | up | 11.88 | 0.004 | TuCCE43: Carboxyl/cholinesterase |
| CCE | tetur16g02380 | up | 11.04 | 0.004 | TuCCE40: Carboxyl/cholinesterase |
| CCE | tetur16g02410 | up | 9.08 | 0.004 | TuCCE42: Carboxyl/cholinesterase |
| CCE | tetur01g08680 | up | 6.27 | 0.007 | TuCCE01: Carboxyl/cholinesterase |
| CCE | tetur29g00930 | up | 3.33 | 0.003 | TuCCE58: Carboxyl/cholinesterase |
| CCE | tetur11g05770 | up | 3.27 | 0.033 | TuCCE34: Carboxyl/cholinesterase |
| CCE | tetur30g01290 | up | 2.76 | 0.009 | TuCCE61: Carboxyl/cholinesterase |
| CCE | tetur17g00300 | up | 2.47 | 0.008 | TuCCE45: Carboxyl/cholinesterase |
| CCE | tetur17g00080 | up | 2.46 | 0.008 | TuCCE44: Carboxyl/cholinesterase |
| CCE | tetur17g00750 | up | 2.40 | 0.009 | TuCCE48: Carboxyl/cholinesterase |
| CCE | tetur17g00350 | up | 2.32 | 0.009 | TuCCE46: Carboxyl/cholinesterase |
| CCE | tetur19g00850 | up | 2.29 | 0.006 | TuCCE49: Acetyl-cholinesterase |
| CCE | tetur11g05760 | up | 2.13 | 0.025 | TuCCE33: Carboxyl/cholinesterase |
| CCE | tetur11g01570 | up | 2.12 | 0.013 | TuCCE31: Carboxyl/cholinesterase |
| CCE | tetur04g06770 | up | 2.11 | 0.009 | TuCCE25: Carboxyl/cholinesterase |
| CCE | tetur207g00010 | up | 2.08 | 0.010 | TuCCE70: Carboxyl/cholinesterase |
| CCE | tetur29g00970 | up | 2.08 | 0.004 | TuCCE60: Carboxyl/cholinesterase |
| **Gene family** | ***T. urticae***  **accession number** | **Regulation** | **Absolute**  **Fold change** | **Corrected**  **p-value** | **Gene name** |
| CCE | tetur01g14180 | down | 30.50 | 0.008 | TuCCE12: Carboxyl/cholinesterase |
| CCE | tetur13g03700 | down | 22.65 | 0.003 | TuCCE39: Carboxyl/cholinesterase |
| CCE | tetur03g00310 | down | 15.69 | 0.003 | TuCCE20: Carboxyl/cholinesterase |
| CCE | tetur11g01500 | down | 13.55 | 0.004 | TuCCE29: Carboxyl/cholinesterase |
| CCE | tetur02g10640 | down | 6.37 | 0.004 | TuCCE18: Carboxyl/cholinesterase |
| CCE | tetur12g03000 | down | 4.38 | 0.003 | TuCCE36: Carboxyl/cholinesterase |
| CCE | tetur35g00210 | down | 4.15 | 0.006 | TuCCE66: Carboxyl/cholinesterase |
| CCE | tetur12g04610 | down | 3.88 | 0.006 | TuCCE38: Carboxyl/cholinesterase |
| CCE | tetur35g00200 | down | 3.84 | 0.006 | TuCCE65: Carboxyl/cholinesterase |
| CCE | tetur12g04600 | down | 3.39 | 0.003 | TuCCE37: Carboxyl/cholinesterase |
| CCE | tetur26g01130 | down | 2.86 | 0.005 | TuCCE55: Carboxyl/cholinesterase |
| CCE | tetur02g14551 | down | 2.20 | 0.005 | TuCCE19: Carboxyl/cholinesterase |
| CCE | tetur03g02700 | down | 2.03 | 0.008 | TuCCE21: Carboxyl/cholinesterase |

* *T . urticae* accession numbers and their corresponding gene sequences can be found at the ORCAE database (<http://bioinformatics.psb.ugent.be/orcae/overview/Tetur>)
