# Supplementary material for: Genome wide gene-expression analysis of facultative reproductive diapause in the two-spotted spider mite Tetranychus urticae
Source: BMC Genomics. 2013 Nov 21;14(1):815. doi: 10.1186/1471-2164-14-815 (PMC4046741; doi:10.1186/1471-2164-14-815)
Supplement: Supplementary file 8 — Additional file 8: Differentially expressed glutathione S-transferases (GSTs) in diapausing T. urticae females. (DOCX 18 KB) [file 12864_2013_5534_MOESM8_ESM.docx]

Additional Table 8

| **Gene family** | ***T. urticae***  **accession number*** | **Regulation** | **Absolute**  **Fold change** | **Corrected**  **p-value** | **Gene name** |
| --- | --- | --- | --- | --- | --- |
| GST | tetur26g01490 | up | 2.24 | 0.017 | TuGSTd11:Glutathione S-transferase, class delta (glutathione S-transferase D1) |
| GST | tetur05g05190 | down | 5.20 | 0.006 | TuGSTm03:Glutathione S-transferase, class mu (glutathione S-transferase) |
| GST | tetur05g05220 | down | 2.90 | 0.010 | TuGSTm06:Glutathione S-transferase, class mu (glutathione S-transferase) |
| GST | tetur05g05180 | down | 2.88 | 0.006 | TuGSTm02:Glutathione S-transferase, class mu (glutathione S-transferase) |
| GST | tetur01g02480 | down | 2.40 | 0.005 | TuGSTd03:Glutathione S-transferase, class delta (glutathione S-transferase) |
| GST | tetur05g05260 | down | 2.18 | 0.023 | TuGSTm09:Glutathione S-transferase, class mu (glutathione S-transferase) |
| GST | tetur01g02320 | down | 2.16 | 0.004 | TuGSTo01:Glutathione S-transferase, class omega (glutathione-S-transferase) |
| GST | tetur01g02500 | down | 2.09 | 0.004 | TuGSTd04:Glutathione S-transferase, class delta (glutathione S-transferase) |
| GST | tetur05g05240 | down | 2.05 | 0.022 | TuGSTm07:Glutathione S-transferase, class mu (glutathione S-transferase) |

* *T . urticae* accession numbers and their corresponding gene sequences can be found at the ORCAE database (<http://bioinformatics.psb.ugent.be/orcae/overview/Tetur>)
