# Supplementary material for: Genome wide gene-expression analysis of facultative reproductive diapause in the two-spotted spider mite Tetranychus urticae
Source: BMC Genomics. 2013 Nov 21;14(1):815. doi: 10.1186/1471-2164-14-815 (PMC4046741; doi:10.1186/1471-2164-14-815)
Supplement: Supplementary file 9 — Additional file 9: Differentially expressed ABC-transporters (ABCs) in diapausing T. urticae females. (DOCX 18 KB) [file 12864_2013_5534_MOESM9_ESM.docx]

Additional File 9

| ***T. urticae* accession number*** | **regulation** | **Fold Change Absolute** | **Corrected**  **p-value** | **Gene name** | **Gene family** |
| --- | --- | --- | --- | --- | --- |
| tetur11g05990 | up | 2.09 | 0.007 | TuABCC-29:ABC-transporter, class C | ABCC |
| tetur23g02452 | down | 7.36 | 0.005 | TuABCC-38:ABC-transporter, class C | ABCC |
| tetur25g01780 | down | 6.87 | 0.011 | TuABCC-35:ABC-transporter, class C | ABCC |
| tetur14g02290 | down | 2.74 | 0.004 | TuABCC-30:ABC-transporter, class C | ABCC |
| tetur14g02320 | down | 2.72 | 0.004 | TuABCC-32:ABC-transporter, class C | ABCC |
| tetur14g02310 | down | 2.65 | 0.004 | TuABCC-31:ABC-transporter, class C | ABCC |
| tetur14g02330 | down | 2.57 | 0.004 | TuABCC-33:ABC-transporter, class C | ABCC |
| tetur03g09880 | down | 2.38 | 0.011 | TuABCC-11:ABC-transporter, class C | ABCC |
| tetur03g09800 | down | 2.32 | 0.012 | TuABCC-10:ABC-transporter, class C | ABCC |
| tetur40g00010 | down | 2.17 | 0.012 | TuABCC-36:ABC-transporter, class C | ABCC |
| tetur17g02510 | up | 3.50 | 0.005 | TuABCG-16:ABC-transporter, class G | ABCG |
| tetur13g02010 | up | 3.36 | 0.015 | TuABCG-11: ABC-transporter, class G | ABCG |
| tetur03g04350 | up | 2.85 | 0.008 | TuABCG-04:ABC-transporter, class G | ABCG |
| tetur02g11270 | down | 2.32 | 0.007 | TuABCG-01:ABC-transporter, class G | ABCG |
| tetur18g00230 | up | 4.52 | 0.033 | TuABCH-13:ABC-transporter, class H | ABCH |
| tetur21g00940 | up | 4.13 | 0.009 | TuABCH-15:ABC-transporter, class H | ABCH |
| tetur01g03530 | up | 2.23 | 0.004 | TuABCH-01:ABC-transporter, class H | ABCH |
| tetur26g02620 | down | 5.60 | 0.003 | TuABCH-16:ABC-transporter, class H | ABCH |
| tetur05g05000 | down | 4.27 | 0.005 | TuABCH-07:ABC-transporter, class H | ABCH |
| tetur36g00240 | down | 2.29 | 0.031 | TuABCH-21:ABC-transporter, class H | ABCH |

* *T . urticae* accession numbers and their corresponding gene sequences can be found at the ORCAE database (<http://bioinformatics.psb.ugent.be/orcae/overview/Tetur>)
