# Supplementary material for: Genome wide gene-expression analysis of facultative reproductive diapause in the two-spotted spider mite Tetranychus urticae
Source: BMC Genomics. 2013 Nov 21;14(1):815. doi: 10.1186/1471-2164-14-815 (PMC4046741; doi:10.1186/1471-2164-14-815)
Supplement: Supplementary file 10 — Additional file 10: Differentially expressed intradiol ring-cleavage dioxygenases (ID-RCDs) in diapausing T. urticae females. (DOCX 18 KB) [file 12864_2013_5534_MOESM10_ESM.docx]

Additional File 10

| **Gene family** | ***T. urticae***  **accession number*** | **Regulation** | **Absolute**  **Fold change** | **Corrected**  **p-value** | **Gene name** |
| --- | --- | --- | --- | --- | --- |
| ID-RCD | tetur13g04550 | up | 4.15 | 0.0110 | n/a:intradiol ring-cleavage dioxygenase |
| ID-RCD | tetur01g00490 | up | 3.24 | 0.0028 | n/a:intradiol ring-cleavage dioxygenase |
| ID-RCD | tetur07g05940 | up | 3.14 | 0.0028 | n/a:intradiol ring-cleavage dioxygenase |
| ID-RCD | tetur07g05930 | up | 3.13 | 0.0028 | n/a:intradiol ring-cleavage dioxygenase |
| ID-RCD | tetur04g08620 | up | 2.93 | 0.0040 | n/a:intradiol ring-cleavage dioxygenase |
| ID-RCD | tetur12g04671 | up | 2.81 | 0.0028 | n/a:intradiol ring-cleavage dioxygenase |
| ID-RCD | tetur28g01250 | up | 2.77 | 0.0042 | n/a:intradiol ring-cleavage dioxygenase |
| ID-RCD | tetur19g03360 | up | 2.77 | 0.0031 | n/a:intradiol ring-cleavage dioxygenase |
| ID-RCD | tetur19g02300 | up | 2.41 | 0.0067 | n/a:intradiol ring-cleavage dioxygenase |
| ID-RCD | tetur44g00140 | up | 2.39 | 0.0082 | n/a:intradiol ring-cleavage dioxygenase |

* *T . urticae* accession numbers and their corresponding gene sequences can be found at the ORCAE database (<http://bioinformatics.psb.ugent.be/orcae/overview/Tetur>)
