# Supplementary material for: Genome wide gene-expression analysis of facultative reproductive diapause in the two-spotted spider mite Tetranychus urticae
Source: BMC Genomics. 2013 Nov 21;14(1):815. doi: 10.1186/1471-2164-14-815 (PMC4046741; doi:10.1186/1471-2164-14-815)
Supplement: Supplementary file 12 — Additional file 12: Differentially expressed antifreeze proteins (AFPs) in diapausing T. urticae females. (DOCX 18 KB) [file 12864_2013_5534_MOESM12_ESM.docx]

Additional File 12

| **Gene family** | ***T. urticae***  **accession number*** | **Regulation** | **Absolute**  **Fold change** | **Corrected**  **p-value** | **Gene name** |
| --- | --- | --- | --- | --- | --- |
| AFP | tetur22g02690 | up | 164.9 | 0.003 | n/a:Hypothetical protein (hypothetical phage protein) |
| AFP | tetur22g02640 | up | 45.67 | 0.003 | n/a:Hypothetical protein (No hits found) |
| AFP | tetur63g00030 | up | 42.89 | 0.003 | n/a:Hypothetical protein (No hits found) |
| AFP | tetur22g02700 | up | 35.39 | 0.003 | n/a:Hypothetical protein (No hits found) |
| AFP | tetur22g02780 | up | 35.39 | 0.003 | n/a:Hypothetical protein (No hits found) |
| AFP | tetur22g02670 | up | 19.44 | 0.004 | n/a:hypothetical phage protein (hypothetical protein) |
| AFP | tetur63g00050 | up | 14.39 | 0.004 | n/a:hypothetical phage protein (hypothetical protein) |
| AFP | tetur22g02810 | up | 9.8 | 0.004 | n/a: Hypothetical protein (No hits found) |
| AFP | tetur283g00030 | up | 9.57 | 0.003 | n/a:Hypothetical protein (No hits found) |
| AFP | tetur22g02790 | up | 8.17 | 0.003 | n/a:phage protein (hypothetical protein) |
| AFP | tetur22g02550 | up | 7.65 | 0.005 | n/a:Hypothetical protein (No hits found) |
| AFP | tetur22g02730 | up | 5.29 | 0.003 | n/a:Hypothetical protein (No hits found) |
| AFP | tetur22g02800 | up | 5.14 | 0.008 | n/a:Hypothetical protein (No hits found) |
| AFP | tetur63g00070 | up | 4.94 | 0.004 | n/a:Hypothetical protein (No hits found) |

* *T . urticae* accession numbers and their corresponding gene sequences can be found at the ORCAE database (<http://bioinformatics.psb.ugent.be/orcae/overview/Tetur>)
