# Supplementary material for: Genome wide gene-expression analysis of facultative reproductive diapause in the two-spotted spider mite Tetranychus urticae
Source: BMC Genomics. 2013 Nov 21;14(1):815. doi: 10.1186/1471-2164-14-815 (PMC4046741; doi:10.1186/1471-2164-14-815)
Supplement: Supplementary file 13 — Additional file 13: Percentage identity/similarity between T. urticae AFP protein sequences. (DOCX 25 KB) [file 12864_2013_5534_MOESM13_ESM.docx]

Additional File 13*

|  | 1 | 2 | 3 | 4 | 5 | 6 | 7 | 8 | 9 | 10 | 11 | 12 | 13 | 14 | 15 | 16 | 17 | 18 | 19 | 20 |
| --- | --- | --- | --- | --- | --- | --- | --- | --- | --- | --- | --- | --- | --- | --- | --- | --- | --- | --- | --- | --- |
| 1. tetur22g02550** |  | 48.2 | 43.3 | 35.5 | 77.1 | 54.9 | 43.8 | 62.4 | 66 | 55.4 | 90.8 | 77.8 | 56.7 | 38.3 | 62.5 | 97.9 | 48.2 | 42.4 | 54.9 | 35.5 |
| 2. tetur22g02640 | 63.8 |  | 36.7 | 40.3 | 50.3 | 46.5 | 35.4 | 66.7 | 51 | 45.2 | 49.6 | 45.1 | 47.3 | 41.5 | 44.4 | 49.3 | 99.2 | 36.2 | 45.3 | 40.3 |
| 3. tetur22g02670 | 55.2 | 44.8 |  | 23.8 | 49.5 | 53.3 | 79 | 43.8 | 45.7 | 54.6 | 44.8 | 49 | 41.4 | 25.2 | 44 | 44.1 | 36.7 | 96.7 | 52.4 | 23.8 |
| 4. tetur22g02690 | 50.4 | 56.9 | 29.5 |  | 37.3 | 33.3 | 26.8 | 43 | 32.7 | 32.7 | 34.8 | 35.9 | 38.3 | 73.7 | 33.8 | 34.5 | 41.1 | 25.2 | 34.6 | 100 |
| 5. tetur22g02700 | 85.6 | 63.4 | 59 | 48.4 |  | 55.6 | 49 | 61.4 | 78.4 | 59.5 | 77.8 | 88.9 | 55.6 | 39.2 | 65 | 76.6 | 49.7 | 48.1 | 56.2 | 37.3 |
| 6. tetur22g02730 | 67.9 | 58.5 | 60 | 40.9 | 69.2 |  | 49.3 | 61 | 54.5 | 69 | 54.9 | 53.1 | 58 | 35.8 | 47.7 | 55.2 | 46.5 | 54.3 | 96.9 | 33.3 |
| 7. tetur22g02740 | 55 | 41.1 | 85.2 | 32.5 | 56.9 | 57.9 |  | 42.6 | 46.2 | 53.2 | 43.8 | 47.1 | 41.4 | 27.8 | 41.7 | 44.1 | 35.9 | 77.6 | 50.7 | 26.8 |
| 8. tetur22g02760 | 73 | 74.8 | 51.4 | 52.6 | 70.6 | 68.6 | 49.8 |  | 62.7 | 57.1 | 63.8 | 57.5 | 59.4 | 46.7 | 51.3 | 62.7 | 65.9 | 43.3 | 59.1 | 43 |
| 9. tetur22g02780 | 75.8 | 61.4 | 56.7 | 42.5 | 85 | 66.7 | 54.1 | 70.6 |  | 54.2 | 66.7 | 73.2 | 51.6 | 39.2 | 55.4 | 65.6 | 51 | 45.7 | 54.8 | 32.7 |
| 10. tetur22g02790 | 69 | 54.8 | 66.7 | 40.5 | 73.8 | 82.1 | 63.2 | 66.7 | 67.3 |  | 55.4 | 56.5 | 57.1 | 33.9 | 57.7 | 55.6 | 45.2 | 53.7 | 67.9 | 32.7 |
| 11. tetur22g02800 | 96.5 | 63.1 | 53.8 | 48.9 | 84.3 | 66 | 54.1 | 73 | 75.2 | 69.6 |  | 82.4 | 56.7 | 37.6 | 65 | 90.1 | 48.9 | 42.9 | 55.6 | 34.8 |
| 12. tetur22g02810 | 84.3 | 58.8 | 58.1 | 46.4 | 91.5 | 66.7 | 57.9 | 68.6 | 80.4 | 72 | 86.3 |  | 51 | 39.2 | 63.1 | 77.3 | 44.4 | 48.1 | 53.1 | 35.9 |
| 13. tetur22g03033 | 70.9 | 64.8 | 49.5 | 46.9 | 62.7 | 66 | 49.3 | 69.6 | 62.1 | 64.3 | 68.1 | 63.4 |  | 39.5 | 48.8 | 57 | 46.5 | 40.5 | 57.4 | 38.3 |
| 14. tetur22g03063 | 48.2 | 54.5 | 31.9 | 85.3 | 48.4 | 42.1 | 34.4 | 54.8 | 45.1 | 41.7 | 48.9 | 48.4 | 50 |  | 36.9 | 38 | 41.5 | 26.2 | 32.1 | 73.7 |
| 15. tetur22g03073 | 73.8 | 54.4 | 53.3 | 42.5 | 80 | 63.8 | 52.6 | 61.9 | 70 | 72 | 74.4 | 76.3 | 58.8 | 44.4 |  | 62.1 | 43.8 | 44.7 | 47.1 | 33.8 |
| 16. tetur283g00030 | 98.6 | 62.7 | 55.2 | 47.9 | 85 | 67.3 | 54.5 | 71.8 | 75.2 | 68.5 | 95.1 | 83.7 | 69.7 | 47.9 | 74.4 |  | 47.9 | 42.2 | 54 | 34.5 |
| 17. tetur63g00030 | 63.8 | 99.2 | 44.3 | 56.9 | 62.7 | 57.9 | 42.1 | 74.1 | 61.4 | 53.6 | 62.4 | 58.2 | 64.1 | 54.5 | 53.8 | 62.7 |  | 36.2 | 45.3 | 40.3 |
| 18. tetur63g00050 | 54.8 | 44.3 | 98.1 | 31.4 | 57.6 | 61.4 | 84.3 | 51 | 56.2 | 65.7 | 52.4 | 56.7 | 50.5 | 32.4 | 54.8 | 54.3 | 43.8 |  | 52.9 | 25.2 |
| 19. tetur63g00070 | 67.3 | 57.9 | 59.5 | 40.9 | 69.2 | 98.7 | 57.9 | 66.7 | 67.9 | 81 | 66 | 66.7 | 65.4 | 44.7 | 66.3 | 65.4 | 57.2 | 61.4 |  | 34 |
| 20. tetur63g00090 | 50.4 | 56.9 | 29.5 | 100 | 48.4 | 40.9 | 32.5 | 52.6 | 42.5 | 40.5 | 48.9 | 46.4 | 46.9 | 85.3 | 42.5 | 47.9 | 56.9 | 31.4 | 42.1 |  |

*amino acid sequence identity (green) and similarity (red) between *T. urticae* AFPs were calculated using MatGat

* **T. urticae* accession numbers and their corresponding gene sequences can be found at the ORCAE database (<http://bioinformatics.psb.ugent.be/orcae/overview/Tetur>)
