# Supplementary material for: Genome wide gene-expression analysis of facultative reproductive diapause in the two-spotted spider mite Tetranychus urticae
Source: BMC Genomics. 2013 Nov 21;14(1):815. doi: 10.1186/1471-2164-14-815 (PMC4046741; doi:10.1186/1471-2164-14-815)
Supplement: Supplementary file 16 — Additional file 16: Differentially expressed genes involved in carotenoid synthesis in diapausing T. urticae females. (DOCX 16 KB) [file 12864_2013_5534_MOESM16_ESM.docx]

Additional File 16

| **Gene name** | ***T. urticae***  **accession number *** | **Regulation** | **Absolute**  **fold change** | **Corrected**  **p-value** |
| --- | --- | --- | --- | --- |
| phytoene dehydrogenase/  phytoene desaturase | tetur01g11270 | up | 5.8 | 0.003 |
| phytoene dehydrogenase/  phytoene desaturase | tetur11g04810 | - | - | - |
| phytoene dehydrogenase/  phytoene desaturase | tetur11g04820 | down | 24.45 | 0.005 |
| lycopene cyclase / phytoene synthase | tetur01g11260 | up | 6.21 | 0.004 |
| lycopene cyclase / phytoene synthase | tetur11g04840 | down | 1.83 | 0.001 |

* *T . urticae* accession numbers and their corresponding gene sequences can be found at the ORCAE database (<http://bioinformatics.psb.ugent.be/orcae/overview/Tetur>)
