# Supplementary material for: Genome wide gene-expression analysis of facultative reproductive diapause in the two-spotted spider mite Tetranychus urticae
Source: BMC Genomics. 2013 Nov 21;14(1):815. doi: 10.1186/1471-2164-14-815 (PMC4046741; doi:10.1186/1471-2164-14-815)
Supplement: Supplementary file 17 — Additional file 17: Differentially expressed genes involved in signal transduction in diapausing T. urticae females. (DOCX 23 KB) [file 12864_2013_5534_MOESM17_ESM.docx]

| ***T. Urticae* accession number***** | **Regulation** | **Absolute**  **Fold change** | **Corrected**  **p-value** | **Topblast**** | **Annotation Bogas** |
| --- | --- | --- | --- | --- | --- |
| tetur03g05860 | up | 7,64 | 0,003 | NA | GPCR104:G-protein coupled receptor |
| tetur30g00590 | up | 4,46 | 0,006 | NA | SLS:Titin, Kettin (sallimus, isoform C) |
| tetur27g01360 | up | 3,89 | 0,006 | regulator of g-protein signaling 7-like | regulator of G-protein signaling 7 |
| tetur12g03620 | up | 3,80 | 0,003 | low quality protein: gamma-aminobutyric acid receptor subunit beta-like | TuGABA-01:GABA gated chloride channel, Rdl-homolog |
| tetur05g02770 | up | 3,55 | 0,009 | a chain thermodynamic and structure guided design of statin hmg-coa reductase inhibitors | HMGR:HMG-CoA reductase |
| tetur05g04360 | up | 3,22 | 0,004 | growth differentiation factor 11-like | myo:myostatin |
| tetur22g01550 | up | 3,13 | 0,004 | transforming growth factor-beta receptor type | TeturTGFbR6:TGFbeta receptor type I |
| tetur16g01090 | up | 3,12 | 0,005 | protein kinase c epsilon type | protein kinase C eta type |
| tetur36g00580 | up | 3,03 | 0,003 | gamma-aminobutyric acid receptor isoform 3b | TuGABA-02:GABA gated chloride channel |
| tetur06g03760 | up | 2,83 | 0,003 | excitatory amino acid | iGluR6:Ionotropic Glutamate Receptor |
| tetur05g00270 | up | 2,71 | 0,003 | tachykinin receptor-like | PREDICTED: tachykinin receptor-like protein-like |
| tetur19g02370 | up | 2,71 | 0,006 | hypothetical protein DAPPUDRAFT_330735 | axotactin |
| tetur07g04810 | up | 2,68 | 0,003 | nuclear hormone receptor e75 | E78:Ecdysone-induced protein 78 |
| tetur12g00650 | up | 2,67 | 0,008 | map-kinase activating death domain | MAP-kinase activating death domain |
| tetur19g00930 | up | 2,55 | 0,009 | member ras oncogene family | ras-related protein Rab-32 |
| tetur14g00130 | up | 2,52 | 0,006 | NA | Hypothetical protein |
| tetur11g02070 | up | 2,51 | 0,007 | tribbles log 2 | tribbles homolog 2 |
| tetur41g00280 | up | 2,51 | 0,007 | nuclear receptor | PREDICTED: nuclear receptor coactivator 2 |
| tetur02g01690 | up | 2,50 | 0,007 | diacylglycerol kinase 1-like | Diacylglycerol kinase beta |
| tetur14g02750 | up | 2,49 | 0,028 | type 1 serotonin receptor | 5-hydroxytryptamine receptor 1A |
| tetur23g02110 | up | 2,42 | 0,008 | triple functional domain protein | unnamed protein product |
| tetur22g00780 | up | 2,41 | 0,016 | inactive phospholipase c-like protein partial | unnamed protein product |
| tetur07g03500 | up | 2,41 | 0,024 | isoform a | myeloid/lymphoid or mixed-lineage leukemia |
| tetur14g02570 | up | 2,39 | 0,005 | regulator of g-protein | regulator of G-protein signaling 19 |
| tetur03g05800 | up | 2,38 | 0,006 | transcription factor sox-17 | sox17:HMG-box transcription factor |
| tetur11g04990 | up | 2,37 | 0,004 | NA | Hypothetical protein |
| tetur04g01970 | up | 2,37 | 0,007 | metabotropic glutamate receptor 1 isoform 2 | GRM1:Metabotropic glutamate receptor homologue, group I mGluR |
| tetur15g00970 | up | 2,34 | 0,004 | myosin-i heavy chain | myosin |
| tetur14g00140 | up | 2,33 | 0,009 | dual 3 -cyclic-amp and -gmp phosphodiesterase 11 | PREDICTED: phosphodiesterase 11A |
| tetur26g00690 | up | 2,32 | 0,013 | run and tbc1 domain-containing | SGSM1:Small G protein signaling modulator 1 |
| tetur40g00260 | up | 2,29 | 0,008 | diacylglycerol partial | DGK2:diacylglycerol kinase zeta homolog |
| tetur07g00110 | up | 2,29 | 0,021 | myosin-i heavy chain | Hypothetical protein (integrin beta subunit) |
| tetur34g00570 | up | 2,28 | 0,003 | serine threonine-protein kinase | serine/threonine-protein kinase LATS1 |
| tetur11g04330 | up | 2,26 | 0,004 | adp-ribosylation factor gtpase-activating protein 2-like isoform 2 | ADP-ribosylation factor GTPase-activating protein 3 |
| tetur15g02730 | up | 2,25 | 0,005 | ankyrin unc44 | UNCoordinated family member |
| tetur06g04400 | up | 2,25 | 0,004 | protein wnt-7b-like | Wnt7:similar to wingless-type MMTV integration site family, member 7B |
| tetur04g03270 | up | 2,25 | 0,006 | guanine nucleotide-binding protein g subunit | guanine nucleotide-binding protein G |
| tetur02g13090 | up | 2,25 | 0,017 | dual specificity phosphatase 3 | Dual specificity protein phosphatase 3 |
| tetur13g00380 | up | 2,25 | 0,003 | growth hormone-regulated tbc partial | PREDICTED: TBC1 domain family member 30-like |
| tetur08g01880 | up | 2,23 | 0,004 | neurotrophic tyrosine kinase receptor precursor | tyrosine protein kinase, putative |
| tetur11g04370 | up | 2,21 | 0,004 | ajuba lim isoform b | limd1:limd1 (LIM domains-containing protein) |
| tetur34g01060 | up | 2,20 | 0,010 | NA | PREDICTED: similar to cAMP-dependent protein kinase type II regulatory chain |
| tetur14g00960 | up | 2,19 | 0,012 | neuropeptide ff receptor 2 | tachykinin receptor-like protein-like (neuropeptide Y receptor, putative) |
| tetur05g01210 | up | 2,19 | 0,010 | niemann-pick c1 | NPC1:Niemann-Pick C1 lysosomal membrane protein |
| tetur15g02440 | up | 2,18 | 0,007 | choline transporter-like protein 2-like | choline transporter-like protein 2 |
| tetur01g01340 | up | 2,18 | 0,006 | ribosomal protein s6 kinase polypeptide 3a | Ribosomal protein S6 kinase, 90kDa, polypeptide 2 |
| tetur06g02220 | up | 2,18 | 0,026 | neuralized pats1 | PREDICTED: hypothetical protein (pats1) |
| tetur06g02260 | up | 2,17 | 0,026 | conserved hypothetical protein | hypothetical protein BRAFLDRAFT_127607 |
| tetur16g02770 | up | 2,15 | 0,022 | cryptochrome 1 | cryptochrome 1 |
| tetur05g05350 | up | 2,15 | 0,005 | phospholipase c at isoform c | Plc21C:1-phosphatidylinositol-4,5-bisphosphate phosphodiesterase beta-4 |
| tetur01g12210 | up | 2,14 | 0,006 | rho gtpase-activating protein 39 isoform 2 | PREDICTED: rho GTPase-activating protein 39-like |
| tetur11g04320 | up | 2,11 | 0,005 | ajuba lim isoform b | ADP-ribosylation factor GTPase activating protein 3 |
| tetur34g00650 | up | 2,10 | 0,003 | NA | cadherin EGF LAG seven-pass G-type receptor 3 precursor |
| tetur21g00290 | up | 2,06 | 0,009 | protein fam13a-like | PREDICTED: protein FAM13A-like |
| tetur11g03300 | up | 2,05 | 0,004 | snf7 domain containing 2 variant 1 | Charged multivesicular body protein 5 |
| tetur14g02820 | up | 2,05 | 0,038 | serotonin receptor | PREDICTED: 5-hydroxytryptamine receptor 1D-like |
| tetur30g01090 | up | 2,04 | 0,007 | plexin domain-containing protein 2-like | Plexin domain-containing protein 2 |
| tetur03g07520 | up | 2,04 | 0,013 | tyrosine protein phosphatase non-receptor | RPTP1:Receptor Protein Tyrosine Phosphatase |
| tetur28g01180 | up | 2,01 | 0,011 | type 1 serotonin receptor | PREDICTED: similar to RHO guanyl-nucleotide exchange factor |
| tetur32g00400 | up | 2,00 | 0,004 | a chain structural studies of metal binding by inositol monophosphatase: evidence for two-metal ion catalysis | inositol monophosphatase 1 |
| tetur11g05800 | down | 2,02 | 0,039 | matricellular protein osteonectin sparc bm- | unnamed protein product (AGAP000305-PA) |
| tetur28g00470 | down | 2,15 | 0,004 | guanine nucleotide-binding protein subunit alpha homolog | guanine nucleotide-binding protein G |
| tetur17g01950 | down | 2,17 | 0,005 | GA18052 | ADP-ribosylation factor GTPase-activating protein 1 |
| tetur01g09220 | down | 2,31 | 0,006 | retinoid x | retinoid X receptor beta |
| tetur35g00400 | down | 2,33 | 0,009 | g-protein coupled receptor 161 | olfactory receptor, family 6, subfamily C, member 3-like (PREDICTED: probable G-protein coupled receptor 21-like) |
| tetur21g00360 | down | 2,38 | 0,005 | isoform b | dysfusion (Neuronal PAS domain-containing protein 4) |
| tetur10g02210 | down | 3,03 | 0,007 | NA | NPF-1:Neuropeptide precursor |
| tetur08g01210 | down | 3,98 | 0,005 | nuclear receptor subfamily 2 group e member 1 | nuclear receptor subfamily 2, group E, member 1 |
| tetur36g00260 | down | 4,27 | 0,005 | daf-12 a6 | HR96-like nuclear receptor h |
| tetur04g08550 | down | 4,93 | 0,008 | NA | Hypothetical protein |
| tetur15g00700 | down | 5,11 | 0,003 | adenosine a3 | Insulin GPCR:Leucine rich repeat G-protein coupled receptor |
| tetur10g00920 | down | 6,42 | 0,009 | lysosome membrane protein 2-like | unnamed protein product (scavenger receptor class B member 1 isoform 3) |
| tetur18g01690 | down | 7,79 | 0,007 | angiopoietin 4 | angiopoietin-4 precursor |

* *T . urticae* accession numbers and their corresponding gene sequences can be found at the ORCAE database (<http://bioinformatics.psb.ugent.be/orcae/overview/Tetur>)

** Topblast is the blast result generated by Blast2go with cutoff E-value=1e^-15^
