# Supplementary material for: Genome wide gene-expression analysis of facultative reproductive diapause in the two-spotted spider mite Tetranychus urticae
Source: BMC Genomics. 2013 Nov 21;14(1):815. doi: 10.1186/1471-2164-14-815 (PMC4046741; doi:10.1186/1471-2164-14-815)
Supplement: Supplementary file 14 — Additional file 14: Alignment of T. urticae antifreeze proteins (AFPs) with those of T. molitor (pdb: 1L1I_A) and D. canadiensis (AFP1A and AFP7 (GenBank acc. numbers AAB94303.1 and AAF86362.1, respectively). AFP protein sequences, without their predicted signal peptides, were aligned using MUSCLE [47] and the resulting alignment was graphically edited using BioEdit version 7.2 [144]. (PDF 78 KB) [file 12864_2013_5534_MOESM14_ESM.pdf]

```
                             10        20        30        40        50        60        70        80        90       100
                    ....|....|....|....|....|....|....|....|....|....|....|....|....|....|....|....|....|....|....|....|
D. canadensis AFP1A ----------------------QCTGGSDCRSCTVSCTDCQNCPNARTACTR------------------------------SSNCINALT---------
D. canadensis AFP7  VTVNSFLIISVVLMFLCHEYYAVCTGGGNCSACTTACTNCVNCPNALLACTD------------------------------STKCMKAVT---------
T. molitor 1L1I_A   ----------------------QCTGGADCTSCTGACTGCGNCPNA-VTCTN------------------------------SQHCVKANT---------
tetur22g02690       ------------------MTPVDSYGNTNCKNC-VNCTDCADCQDC-ENCAN------------------------------------------------
tetur63g00090       ------------------MTPVDSYGNTNCKNC-VNCTDCADCQDC-ENCAN------------------------------------------------
tetur22g03063       ------------------MTSIDSYGNTNCKNC-VNCTDCTDCQDC-NDCKN------------------------------------------------
tetur22g03073       -----SGGSHGSPDSDGNINCVNCKGCTNCTNC-INCINCHGCEDC-SDCTR-----------------CVNCSGNSSILTDCSNCVNCSS---------
tetur22g02780       -----------STDSDGNTNCVNCTGCTNCSNC-INCTNCHGCESC-SDSTN------------------------------CHNSINCAN---------
tetur22g02700       -----------STDSDGNTNCVNCTGCTNCSNC-INCTNCHGCESC-SDSTN------------------------------CRNSRNCAS---------
tetur22g02810       -------------DSNGNVNCVNCTGCTNCVNC-INCTNCHGCESC-SDSTR------------------------------CRNSRNCAS---------
tetur22g02550       -----------STDSNGNVNCVNCTGCTNCVNC-INCTNCHGCESC-SDSTN------------------------------CRSSRNCTS---------
tetur283g00030      -----------STDSNGNVNCVNCTGCTNCVNC-INCTNCHGCERC-SDSTN------------------------------CRSSRNCTS---------
tetur22g02800       -------------DSNGNVNCVNCTGCTNCVNC-INCTNCHGCESC-SDSTR------------------------------CRNSRNCAS---------
tetur22g02760       --------------SDGNTNCVNCVGCTNCANC-INCTNCHDCANC-TDSTN------------------------------CSNSRNCES---------
tetur22g02640       ---------------DGNTNCVNCVGCTNCSNC-INCTNCHDCANC-TDSEN------------------------------------------------
tetur63g00030       ---------------DGNTNCVNCVGCTNCSNC-INCTNCHDCANC-TDSEN------------------------------------------------
tetur22g03033       ------------TDSDGNQNCVNC-------NC-VNCTNCHGCQNC-KDCTD------------------------------CTNCND------------
tetur22g02740       -------TPSTGSGSDGNQNCVNCVNCTNCINC-TNCTNCHGCQNC-EDCTNGISLRYCKNSRDCNRCDVCTCCFNCTDCVYCTNCRNCSNSSNCIDSTD
tetur22g02670       -----------SDGSDGNQNCINCVGCTNCANC-INCTNCHGCQNC-ADCTNGTSLQNCKNSTDCNRCANCIYCLYCTDCVGCTNCRNCSNSSNCTDSTD
tetur63g00050       -----------TDGSDGNQNCINCVGCTNCANC-INCTNCHGCQNC-ADCTNGTSLQNCKNSTDCNRCANCTYCLYCTDCVGCTNCRNCSNSSNCTDSTD
tetur22g02790       -----SGGSDGSPDSDGNINCVNCVGCTNCANC-VNCTNCHGCKNC-NDCTN------------------------------CTNCKNCDN---------
tetur22g02730       -------------SDGNQNCINCVGCTNCANC-INCTNCHNCANC-NDCTN------------------------------CKDCRNCDN---------
tetur63g00070       -------------SDGNQNCINCVGCTNCANC-INCTNCHNCANC-NDCTN------------------------------CKDCRNCDD---------

                            110       120       130       140       150       160       170       180       190       200
                    ....|....|....|....|....|....|....|....|....|....|....|....|....|....|....|....|....|....|....|....|
D. canadensis AFP1A ---------CTDSYDCHNAETCTRSTNCYKAKTCTGSTNCYEA----------------------------TACTDSTG---------CP----------
D. canadensis AFP7  ---------CTRSTKCNKAVTCTNSSDCYKAVTCTGSTNCYKAKSCTG---------------STNCYEATTACVNSTG---------CPP---------
T. molitor 1L1I_A   ---------CTGSTDCNTAQTCTNSKDCFEANTCTDSTNCYKA----------------------------TACTNSSG---------CPGH--------
tetur22g02690       ------------CTGSKNCTNCVNCSNCDDCANCSGSKNCSHCDN------------------CTRCNDC-EKCNTRKD---------CAHRTSLTDCQC
tetur63g00090       ------------CTGSKNCTNCVNCSNCDDCANCSGSKNCSHCDN------------------CTRCNDC-EKCNTRKD---------CAHRTSLTDCQC
tetur22g03063       ---------CANCTGSRNCTNCVNCSNCEDCANCTGSKNCVDCAN------------------CIQCTNC-QKCANRVN---------CANRTGLTNCQC
tetur22g03073       ---------CTNCSDCKNSSSCTDSKNCNDSSSLTNCTNCSNCTSCTK------------CTDCQNCDSC-TNVQNSAN---------CKSRTGCSHCQC
tetur22g02780       ---------CTDCKDCKNCNDCTASGNCEGSRNCTTCTYCSNCADCTSSRNCSDCANCTACKDCQGCSNC-TTCTNSSN---------CKNRTGCTNCQC
tetur22g02700       ---------CTDCSDCKNCASCTASKNCNDCRSCTSCTNCSNCANCTNSRNCSDCTNCTKCTDCQRCTNC-TNVKNSAN---------CLNRTDCTNCQC
tetur22g02810       ---------CTDCSDCKNCASCTASKNCNDSRSCTNCTNCSNCTNCINSRNCSDCTNCTKCTDCQRCTNC-TNVKNSAN---------CTNRTGCTNCKC
tetur22g02550       ---------CTDCSDCKNCTSCTDAKNCNDSRSCTNCRNCSNCASCTN------------CRDCQRCTHC-TNVRNSAN---------CMNRTDCTNCKC
tetur283g00030      ---------CTDCSDCKNCTSCTDAKNCNDSRSCTNCRNCSNCASCTN------------CRDCQRCTNC-TNVRNSAN---------CMNRTDCTNCKC
tetur22g02800       ---------CTDCSDCKNCTSCTDSKNCNDSRSCTNCKNCSNCASCTN------------CADCQRCTNC-TNVKNSAD---------CMDRTGCTNCKC
tetur22g02760       ---------SIDCQRCANGINCVGCSDCADCTGCRNCSNCANCSQCTG---------------CQRCANC-TNCTNCKD---------CANRTGLTNCQC
tetur22g02640       ------------CSNSRNTANSNGCQNCANCTYCVSCSDCADCASSKG---------------CANCANC-TNCTNCRN---------CANRANLSDTQC
tetur63g00030       ------------CSNSRNTANSNGCQNCANCTYCVSCSDCADCASSKG---------------CANCANC-TNCINCRN---------CANRANLSDTQC
tetur22g03033       ---------CRDCQRCANSTDCLSCGDGADCKNCRNCSNCANCRDCID---------------CSNCANC-NRITDSKG---------CADRTDCTQCQC
tetur22g02740       GSYLRYCKTCKDCKRCDNCNNCLRCKDCIYCTDCVYCSDSSNCEDCTNGSYLQNCKTCTDCKRCSGCKDC-LRCSNCANCENCVDAANCNNRTDCTNCQC
tetur22g02670       GSYLQNCKNCKDCKRCSNCANCLRCKDCSNCTDCVYSSDCQNCIDCTNCSYLQNCKTCTDCKRCNNCQDC-LRCSGCNNCKNCVDSANCNDRTDCKNCHC
tetur63g00050       GSYLQNCKNCKDCKRCSNCANCLRCKDCSNCTDCVYSSDCQNCIDCTNCSYSQNCKTCTDCERCNNCQDC-LRCSGCNNCKKCVDSENCNNRTDCKNCHC
tetur22g02790       ---------CRDCQRCTNCPNCLSCEDCSDCKNLRNCSNCANCRECINCSGCKNCENCTDCKRCSNCSDC-LRCTDCKNCTSCEDSANCNNRTDCKNCQC
tetur22g02730       ---------CRDCQRCADCTKCQGCDDTVGSKKCRDCSDCSNCRDCINCSRCRNCESCTDCQRCANCANC-LRCSDCRDCTRCTDSQRCADRTDCKNCQC
tetur63g00070       ---------CRDCSRCADCTKCEGCDDTVGSKKCRDCSDCSNCRDCINCSRCRNCESCADCQRCANCANC-LRCSDCRDCTRCTDSQRCADRTDCKNCQC
```
